# Interests, Motives, and Psychological Burdens in Times of Crisis and Lockdown: Google Trends Analysis to Inform Policy Makers

**DOI:** 10.2196/26385

**Published:** 2021-06-01

**Authors:** Dominik Rotter, Philipp Doebler, Florian Schmitz

**Affiliations:** 1 Department of Psychology University of Duisburg-Essen Essen Germany; 2 Statistical Methods in Social Sciences TU Dortmund University Dortmund Germany

**Keywords:** coronavirus, Google Trends, infodemiology, infoveillance, pandemic, information search, trend, COVID-19, burden, mental health, policy, online health information

## Abstract

**Background:**

In the face of the COVID-19 pandemic, the German government and the 16 German federal states implemented a variety of nonpharmaceutical interventions (NPIs) to decelerate the spread of the SARS-CoV-2 virus and thus prevent a collapse of the health care system. These measures comprised, among others, social distancing, the temporary closure of shops and schools, and a ban of large public gatherings and meetings with people not living in the same household.

**Objective:**

It is fair to assume that the issued NPIs have heavily affected social life and psychological functioning. We therefore aimed to examine possible effects of this lockdown in conjunction with daily new infections and the state of the national economy on people’s interests, motives, and other psychological states.

**Methods:**

We derived 249 keywords from the Google Trends database, tapping into 27 empirically and rationally selected psychological domains. To overcome issues with reliability and specificity of individual indicator variables, broad factors were derived by means of time series factor analysis. All domains were subjected to a change point analysis and time series regression analysis with infection rates, NPIs, and the state of the economy as predictors. All keywords and analyses were preregistered prior to analysis.

**Results:**

With the pandemic arriving in Germany, significant increases in people’s search interests were observed in virtually all domains. Although most of the changes were short-lasting, each had a distinguishable onset during the lockdown period. Regression analysis of the Google Trends data confirmed pronounced autoregressive effects for the investigated variables, while forecasting by means of the tested predictors (ie, daily new infections, NPIs, and the state of economy) was moderate at best.

**Conclusions:**

Our findings indicate that people’s interests, motives, and psychological states are heavily affected in times of crisis and lockdown. Specifically, disease- and virus-related domains (eg, pandemic disease, symptoms) peaked early, whereas personal health strategies (eg, masks, homeschooling) peaked later during the lockdown. Domains addressing social life and psychosocial functioning showed long-term increases in public interest. Renovation was the only domain to show a decrease in search interest with the onset of the lockdown. As changes in search behavior are consistent over multiple domains, a Google Trends analysis may provide information for policy makers on how to adapt and develop intervention, information, and prevention strategies, especially when NPIs are in effect.

## Introduction

On March 11, 2020, the World Health Organization (WHO) declared the COVID-19 outbreak a pandemic, as almost all countries of the world were affected [1]. One month earlier, the WHO announced the official names of this coronavirus and the disease it causes as SARS-CoV-2 and COVID-19, respectively [2]. As of October 3, 2020, there have been 296,958 reported cases in Germany [3] and 34,495,176 reported cases worldwide [4].

With increasing case numbers and death counts comes the necessity to monitor and slow the further spread of the pandemic [5]. There is strong evidence that human-to-human transmission is the reason the initial outbreak in Wuhan, China, became global; thus, it is imperative to prevent secondary infections [6] (eg, by means of restricting air traffic [7] and other nonpharmaceutical interventions [NPIs]) in the absence of a vaccine [8]. NPIs are used to pursue two strategies—mitigation and suppression of further spread—which can be achieved by a combination of multiple population-wide measures, such as social distancing by the entire population; home isolation and quarantine of cases and their household members; banning mass gatherings, and closing schools and universities [8,9]. The implementation of these measures seems to slow the spread of the pandemic, thus preventing thousands of deaths [9,10]. As data for monitoring disease spread is difficult to obtain in the early stages of a pandemic, the use of internet data has become increasingly valuable, with the emergence of two research disciplines: infodemiology and infoveillance [11]. Although the former is a research discipline and methodology studying the determinants and distribution of health information [12], the latter is defined as the longitudinal tracking of infodemiology metrics with the goal of surveillance and trend analysis [13]. Both can help policy makers in developing prevention and information strategies. As information influences knowledge, behavior changes, and health outcomes [11,14], the internet can be used as a handy tool to monitor public search behavior [15-17] or public awareness [18] of health care issues, allowing policy makers to quantify knowledge translation gaps [12], identify misleading information circulation [19,20] or possible emerging local outbreaks [21], assess people’s needs [22], derive suitable interventions [23-25], and develop enhanced communication strategies especially for at-risk subpopulations [26].

To inform policy makers about crisis-related developments, researchers have examined changes in search behavior during the COVID-19 pandemic [27,28]. Google Trends data was used to monitor public interest in the pandemic from January to March 2020 by conceptualizing the demand for information as a single search term (“coronavirus”), showing worldwide peaks in people’s interest at the end of January as well as shortly after COVID-19 was declared a pandemic. Pearson correlations indicated a moderate to high relationship between different countries regarding search behavior [29], although some studies (eg, Hu et al [18]) suggest differences during the first peak between countries and even various subregions of countries. Typically, announcements of the first local COVID-19 case led to a widespread increase in search interest as people sought information about the COVID-19 pandemic [30]. There is evidence, though, that COVID-19 terms trending in Italy elicited differential peaks, showing that regions most heavily affected in terms of infections and deaths were not always the first to display changes in search interest [19], as local and international events are capable of influencing public interest [31]. However, the number of recent COVID-19 infections and deaths is associated with changes in public interest, which becomes apparent by maximum correlations when a lead of approximately –11.5 days is applied [32]. This lagged nature of relationships has also been found for other COVID-19–related search tags (eg, the frequency of search queries for loss of smell [33,34] and taste [34,35] as key symptoms of COVID-19, or public interest in “pneumonia” [36] or “high temperature”, “cough”, and “diabetes” [37], as well as “insomnia”, a key signal for mental distress [38]). The positive relationship seems to be consistent across different social media platforms and search engines, such as Baidu and Sina Weibo Indices [36], as well as with other diseases (eg, the flu outbreak in 2004 [12]). However, Mavragani [39], who used Google Trends data with the search tag “coronavirus” to study European countries (ie, Italy, Spain, France, Germany, and the United Kingdom) found moderate to high positive Pearson correlations between search queries and the number of total and daily new cases for all countries except Italy. An in-depth analysis of multiple time frames indicated a declining trend of the Pearson correlation coefficient over time, highlighting an early interest phenomenon. Overall, the increase of attention is typically short-lived [18,29], even when accompanied by NPIs issued by policy makers [30].

Given that Google Trends data can indicate population health literacy, Google Trends queries for “wash hands” and “face mask” were found to correlate with a lower spreading rate of COVID-19 cases in 21 countries: the logarithmic increase of case numbers correlated negatively with the number of days with high search volume [40]. Google Trends data could moderately predict new cases, although predictions were not found to be very precise [41]. Nonetheless, COVID-19 symptoms showed some predictive value, especially when virus diffusion was monitored by predicting the number of deaths [42]. Even real-time forecasting of the COVID-19 outbreak has been shown to be possible [43,44].

However, changes in search behavior are not only limited to disease symptoms (eg, “anosmia” [45]). Previous research has similarly shown that COVID-19 has affected the social, economic, and psychological well-being of humanity, pervading broad domains of social and daily life, such as COVID-19 awareness [29,32], psychological stress (eg, “anxiety”, “depression”, “therapy” [46]), food supply (eg, “sourdough”, “restaurants”, “baking” [46]), economic stressors (eg, “mortgage”, “homeloan”), social stressors (eg, “divorce”, “liquor”), and treatment seeking (eg, “cognitive therapy”, “counselling” [25]), as well as quarantine and even conspiracy theories [30]. An analysis of Twitter data identified four main domains of interest for people: the origin of the virus, its sources, its impact on people, countries, and economies, as well as ways of mitigating the risk of infection. The latter suggests that Twitter users are keen to learn and also share their knowledge with friends and followers, while also discussing the negative consequences of the COVID-19 pandemic and its emotional and psychological impact [22]. Further research found changes in stress-related and food-related searches, indicating that the outbreak may have had a significant impact on both people’s stress levels and daily routines [46].

Despite a growing body of research studying search behavior regarding COVID-19 and its symptoms in conjunction with infection and death counts, there seem to be limited research activities targeting the effects of the issued NPIs. One early study used Google Trends data from January to March 2020 to examine the impact of the pandemic and the issued NPIs on people’s concerns, indexed by changes in search frequency about mental health issues as well as financial and work-related consequences. They found an increase in financial and work-related queries, indicating that people are well aware of further consequences, such as job loss. There is also some evidence that queries for “depression” and “suicide” decreased immediately after the announced pandemic, but increased shortly after the first wave [25]. Another study stressed the potential damage to a population’s well-being as a consequence of the lockdown. It used Google Trends data to test for changes in people’s well-being and found a substantial increase in search intensity for boredom in European countries and the United States, which was accompanied by a higher search volume for loneliness, worry, and sadness, while searches for stress, suicide, and divorce declined [47].

Since previous research has shown that the COVID-19 pandemic has resulted in changes in public interest [18,29,30] beyond the expected search behavior regarding COVID-19–related symptoms [22], we conducted this study to investigate the effects of the pandemic and the corresponding lockdown using a broad set of psychological domains and interests. As Google Trends data can function as a proxy for psychological states and behaviors [46], search interests may indicate changes in social and daily life. Our 27 domains of interest range from COVID-19 restrictions to online shopping, sexual interests, and psychosocial impact. The German government and the 16 German federal states issued a cluster of NPIs to decelerate the spread of COVID-19. These measures comprised, among others, social distancing, the temporary closure of shops and schools, and banning large public gatherings and meetings with people not living in the same household [5]. As prior studies suggested, it seems fair to assume that all measures heavily affected social life and psychological functioning [25,46-48]. Thus, we expected significant changes in search behavior during the emerging pandemic and the subsequently issued lockdown period for all observed domains. Further, daily new infections were tested as a predictor, given that infection and death counts have been shown to be associated with an increase in public interest [32,35]. Additionally, the state of the national economy was investigated as a predictor, as it may affect people’s employment situation and related psychological anticipations, worries, and the need to search for COVID-19–related information. In turn, changes in search queries and their relevant predictors can be used to inform policy makers, allowing them to tailor information and health prevention strategies [23-25].

## Methods

### Psychological Domains and Indicators of Interest

As preregistered [49], we planned to examine 27 psychologically meaningful domains that may possibly have been affected by the COVID-19 pandemic, the NPIs, or associated economic developments. For each domain, at least three search terms were selected as indicators. For instance, the domain “pandemic disease” comprised “coronavirus”, “covid sars”, or “covid-19” as Google search tags. Descriptions of all domains are given in Table 1. For all search terms, refer to Multimedia Appendix 1.

**Table 1:** All 27 domains and their relevant description.

Domain	Description
Pandemic disease	People’s interest in the virus and the disease it causes
Health care institutions	German and international institutions as well as experts providing information on the pandemic
Political leaders	Names of leading German politicians whose responsibilities comprise dealing with the pandemic
Infection	Information concerning the rapidity and method of virus spread
Symptoms	Possible symptoms experienced by patients with COVID-19 indicating a virus infection
Information seeking	Sources and methods people could use to obtain an overview of the development of the pandemic
Testing	Questions pertaining to the capacities of testing, including locations and general opportunities to have oneself tested
Hand hygiene	Possibilities of keeping one’s hands virus-free
Mask	Interest in the different types of masks, their effectiveness, and the current regulations on wearing a mask
Disinfectant	Possible substances used for disinfection and recommendations for appropriate disinfection
Convenience goods	Groceries or household goods that were nearly sold out due to panic buying and hoarding
Vaccination	Interest in vaccination possibilities and fear of a compulsory vaccination
Parenting and childcare	Challenges parents have to deal with during the pandemic
COVID-19 restrictions	Information about the current restrictions and rules of conduct
COVID-19 relaxations	Information about current and future possibilities and relaxations of the issued restrictions
Economic impact	Economic shortcomings and development as consequences of the COVID-19 restrictions
Sexual interest	Changes in sexual interest due to the COVID-19 crisis
Social life	Possibilities for people to socialize while following COVID-19 restrictions such as social distancing
Homeschooling	Information about new ways of educating students at home while schools were closed
Business communication	Different ways for employees to stay in contact with their colleagues and to be able to work from home
Hobbies and sports	Activities that became popular during the lockdown as they could be done without breaking restrictions
Renovation	Renovation activities that could be done at home during the pandemic
Online shopping	Names of common online sellers
Dispatching	Terms and information about the shipment of items
Psychosocial impact	Negative consequences that arose due to the COVID-19 restrictions
Conspiracy theories	Several conspiracy theories related to the source and spread of the pandemic
Government support	Terms and information about financial aid due to closures and severe restrictions

### Retrieving Google Trends Data

Each domain was entered in the Google Trends database to reveal the number of Google search queries for a given time period and geolocation using the *gtrends* package [50] for the statistical language *R* [51]. Before downloading, we thoroughly studied the framework for using Google Trends data, which highlights the choice of region, time period, and geolocation [11]. As Google allows one to set different geolocations at the country and federal state level within a specified period, we retrieved Google Trends data separately for each search tag for 17 geolocations (ie, Germany and its 16 federal states) for the period from January 6 to September 1, 2020. We chose this period to allow for a sufficient period before the first cases occurred in Germany as a baseline estimate. By default, Google standardizes the queries per day by the maximum of queries within the given period, multiplying all values by 100. High interest in a query is expressed by 100, whereas minor interest or missing data on queries is expressed by 0. Values below 1 were recoded as 1.

We retrieved Google Trends data for all domains for all 17 geolocations except for 32 search tags falling into 16 domains. An overview of the domains and search tags for which we could retrieve Google Trends data is provided in Multimedia Appendix 1. For the analysis, we used only keywords available for all federal states. As there were differences between the retrieved time series for the geolocation “DE” for Germany and the aggregated time series across all federal states, we decided to use the aggregated time series for further analysis.

### Retrieving Daily New Infections Data

As the Robert Koch Institute (RKI), a German federal government agency and research institute responsible for disease control and prevention, issued daily situation reports concerning COVID-19 [52] even before the official declaration of the pandemic, we made use of the online dashboard application programming interface (API) to retrieve daily data, comprising infection and death counts for each federal state as well as Germany as a whole from January 6 to September 1, 2020. Maps of the German federal states together with 7-day incidence rates are provided in Multimedia Appendix 2, along with summary statistics for, for example, population density, cumulative infections, and incidence rates in Multimedia Appendix 3.

### Retrieving Economic Data

The truck toll mileage index (TTMI) provides an early and sensitive day-to-day proxy for the state of industrial production. It is computed by using process data of the toll system for trucks in Germany [53]. According to the German Federal Statistical Office (Statistisches Bundesamt), the TTMI can serve as a highly accurate indicator of the economic state with a lag of 5 to 9 days. We retrieved the data for the period from January 6 to September 1, 2020.

### Retrieving NPI Data

In the face of the COVID-19 pandemic, the German government and its federal states issued 14 NPIs to curb the spread of COVID-19. The measures included limitations regarding leaving home without reason (ie, only for grocery shopping or a doctor’s appointment), recommendations to keep social distance (>1.5m), the obligation to wear a mask in stores and when using public transport, closure of nonessential shops, closure of hairdressers and close contact services (eg, massage studios), closure of nonfood shops (eg, bicycle and hardware stores), closure of zoos, the prohibition of public demonstrations; closure of schools, playgrounds, kindergartens, and day care facilities; closure of religious institutions, and prohibition of meeting in public with persons not belonging to the same household. The Leibniz Institute for Psychology (ZPID) provided coded daily data for all relevant NPIs with their onset, separated by federal state, from March 8 to June 26, 2020 [5]. We will subsequently refer to this period as the lockdown period.

### Data Reduction and Preprocessing

Within each domain, a time series factor analysis (TSFA) [54] was used for Germany to test if one hypothesized dominant factor accounts for the covariation of indicators in the respective domain. First, we extracted the moving average for 7 days (the so-called trend) for all search terms for a domain. Second, we z-standardized the time series to attain loadings, which can be interpreted as correlations (–1≤λ≤1), and estimated the factor analysis with a single assumed factor. We used only the trend information of time series as it represents the long-term process and behavior of the time series [55]. Indicator variables were selected when they had positive loadings on the first factor, thereby iteratively excluding indicators with negative loadings. We excluded 20 search terms falling into 10 domains (Multimedia Appendix 1).

For all domains, the scree plots resulting from the selection process are presented in Multimedia Appendix 4. Next, factor scores were calculated using the loadings on the first factor as weights to aggregate a single time series for each keyword per federal state, retrieving a mean time series for each domain. We then deseasonalized the time series for each domain, supposing an additive model with weekly seasonality for every time series.

Since the NPIs were issued at different times, we used TSFA to identify factors that describe their covariation. We differenced every time series, so they corresponded with daily change scores, prior to computing the TSFA. Scree plots could be interpreted as indicating a two- or three-factorial structure. The two-factor model could be more consistently interpreted, yielding one factor of “regulation of outdoor activities” (NPI Outdoor) and another factor of “regulation of social life” (NPI Social). Corresponding with the selection criteria applied to Google Trends data, we excluded indicator time series with negative loadings. We calculated factor scores using the loadings as weights to aggregate single time series for each NPI, resulting in a single mean time series for each NPI factor. Details concerning indicators and their respective loadings are given in Table 2.

**Table 2:** TSFA loadings for the two extracted NPI factors, regulation of outdoor activities (NPI Outdoor) and regulation of social life (NPI Social), for aggregated time series data^a^.

NPI	Regulation of outdoor activities (NPI Outdoor)	Regulation of social life (NPI Social)
Prohibition to leave home	N/A^b^	.91
Social distancing	.31	.74
Rule to wear a mask in public	.06	N/A
Closure of nonessential shops	.28	N/A
Closure of hairdressers and close contact services	.02	.58
Closure of nonfood shops	.39	.22
Closure of zoos	.99	N/A
Prohibition of public demonstrations	.32	N/A
Closure of schools	.41	.05
Closure of religious institutions	.67	.05
Prohibition to meet with nonhousehold members	N/A	.73
Prohibition to meet with more friends	.14	.69
Closure of shops	.93	N/A
Closure of kindergartens and day care	.48	.10

^a^Indicators with negative loadings were omitted from the factor scores. NPI: nonpharmaceutical intervention. TSFA: time series factor analysis.

^b^N/A: not applicable.

### Visual Inspection and Change Point Analysis

Each aggregated time series per domain and federal state was carefully screened in conjunction with the time series for daily new infections, as well as the economic state and the two NPI factors “regulation of outdoor activities” and “regulation of social life.” As we assumed structural breaks, indicating a major change in the underlying processes of the time series, we proceeded with a change point analysis with an intercept-only model to identify changes in the level for the lockdown period [56].

### Time Series Regression Analysis

For regression analysis, we conceptualized search behavior over time for each federal state as a criterion variable in linear mixed models, testing intercept-only models to identify a possible hierarchical structure as it would be indicated by a substantial intraclass correlation (ICC). Since there were no meaningful ICCs (all ICC<0.05, as preregistered), we proceeded with models for Germany only. We used daily new infections, the NPI factors NPI Outdoor and NPI Social, as well as the TTMI as predictors. Criterion and predictor variables were z-standardized before linear modelling. To account for the autocorrelation in time series, we used an autoregressive integrated moving average (ARIMA) approach to fit the models [55,57]. ARIMA models are a form of generalized least squares models with three parameters, namely *p*, *d,* and *q*. The *p* parameter denotes the order of the autoregressive part AR(*p*). In an autoregressive model, the values in a time series Y_t_ are predicted by its lagged values Y_t-p_. For a lag of 1, the AR(1)-model is equal to equation 1.

*Y*_t_ = *β*_0_ + *β*_1_*Y*_t-1_ + ε_t_ (**1**)

For the moving average part, the parameter *q* denotes the order of previous error values ε_t-_*_q_* used to predict the time series *Y*_t_. An MA(*1*)-model is equal to equation 2.

*Y*_t_ = *β*_0_ + *β*_1_ ε_t-1_ + ε_t_ (**2**)

The *d* parameter stands for “integrated” and denotes the order of differencing that was applied to the time series before an autoregressive moving-average (ARMA) analysis. Differencing is used to adjust for seasonality in the time series and can be repeated multiple times. In summary, an ARIMA model with the parameters *p*=1, *d*=1, and *q*=1 with a predictor *X*_1_ and *D*_t_ = *Y*_t_ – *Y*_t-1_ is equal to equation 3.

*D*_t_ = *β*_0_ + *β*_1_*X_1_* + *β*_2_ ε_t-1_ + *β*_3_*D*_t-1_ + ε_t_ (**3**)

In time series regression, predictors are frequently allowed to exert lagged effects. To estimate this lag period, the time parameter is adjusted (shifting the time series) to maximize predictive power. As we were only interested in predicting Google Trends data, we focused on shifting predictors forward from 0 to 21 days. In this case of shifting a time series forward, the value of a time series (*Y*_t_) is replaced by its successive value (*Y*_t+1_), which is called a lead. We refrained from using the Bayesian information criterion [58] for model selection as preregistered [49], since iteratively shifting time series resulted in different combinations of ARIMA parameters, rendering a meaningful interpretation of the respective fit values impossible. Therefore, we selected the lag period for which the predictor exerted a maximal correlation with the criterion variable. The cross-correlation functions for all relevant predictors (ie, daily new infections, NPI factors NPI Outdoor and NPI Social, TTMI) are provided in Multimedia Appendix 5.

All statistical analyses were performed using the statistical language *R* [51] with the package *strucchange* [56] for identifying structural breaks and the package *lme4* [59] for linear modeling.

## Results

### Cross-Correlation Between Domains

**Figure 1:**
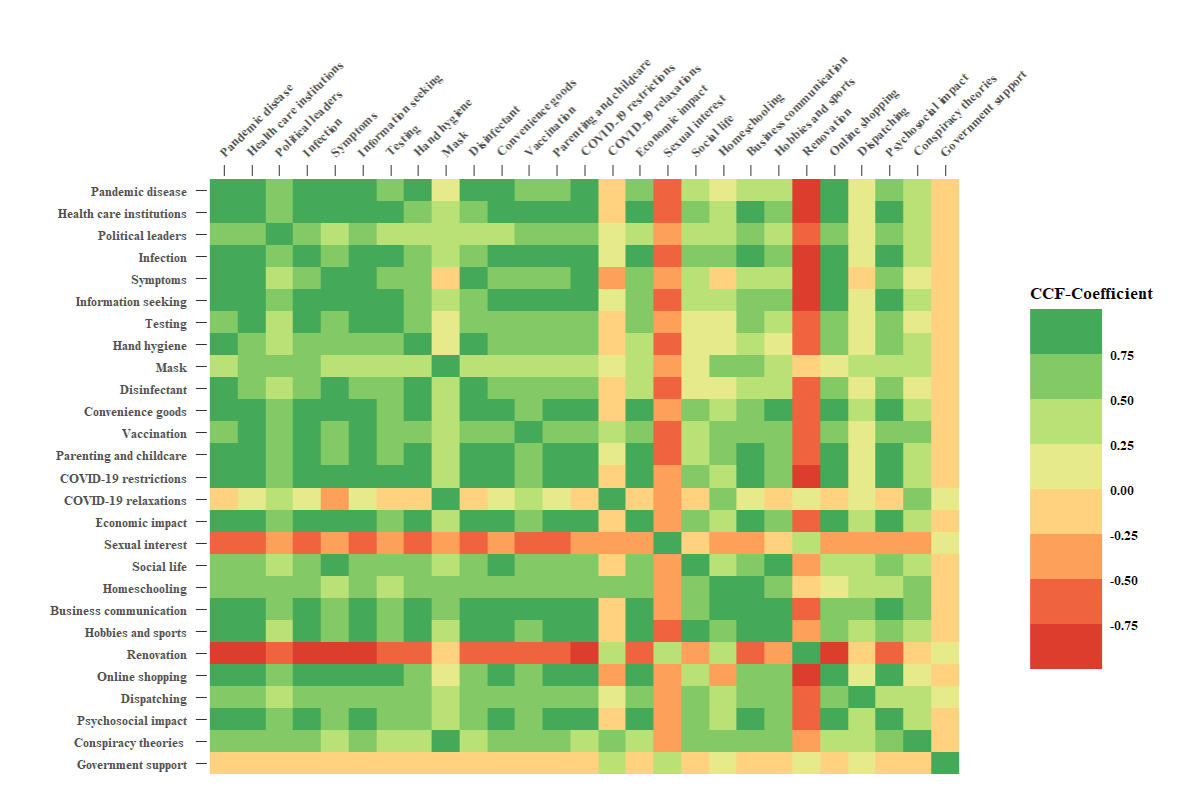
Heat map of cross-correlations for all 27 domains for Germany only. The lower triangle shows concurrent cross-correlations. The upper triangle shows maximal correlations obtained when the time series of one domain was shifted across a range from 0 to 21 days relative to the time series of the corresponding other domain. CCF: cross-correlation function.

Search interests for all 27 domains were moderately to highly correlated (Figure 1, lower triangle). Exceptions can be noticed for the domains *parenting and childcare*, *COVID-19 relaxations*, *sexual interest*, *renovation*, and *government support*. To identify possible lagged relations between domains, we shifted one time series in the range from 0 to 21 days. The maximum correlations obtained within this time-lagged range were highly comparable to the concurrent correlation. Hence, the overall pattern of correlations across domains was hardly affected (Figure 1, upper triangle).

### Visual and Change Point Analysis

**Figure 2:**
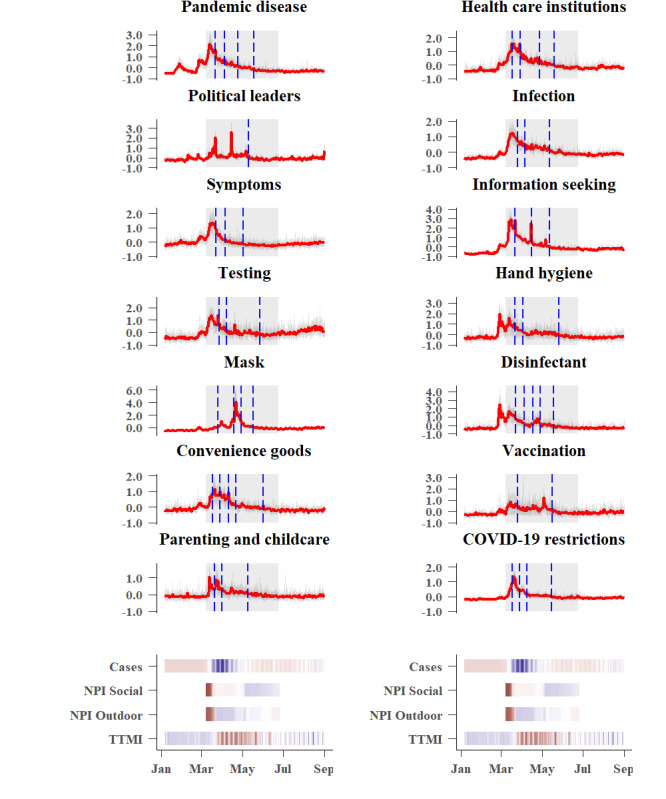
Z-standardized Google Trends for the first 14 domains. The red function indicates the trend for Germany, while the grey functions correspond with the trends for the 16 federal states. The grey shaded area represents the lockdown period in Germany from March 8 to June 26, 2020. Change points, indicating level changes, are represented by the vertical blue dashed lines. The intensity of daily new infections (Cases), countermeasures (NPI Social and NPI Outdoor), and the proxy for the state of economy (TTMI) is plotted below. NPI: nonpharmaceutical intervention; TTMI: truck toll mileage index.

**Figure 3:**
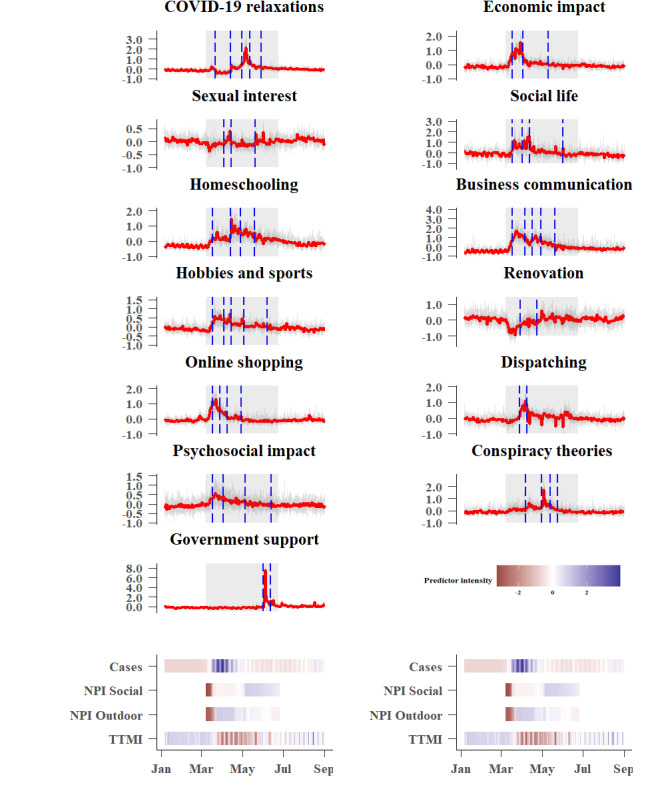
Z-standardized Google Trends for 13 additional domains. The red function indicates the trend for Germany, while the grey functions correspond to the trends for the 16 federal states. The grey shaded area represents the lockdown period in Germany from March 8 to June 26, 2020. Change points, indicating level changes, are represented by the vertical blue dashed lines. The intensity of daily new infections (Cases), countermeasures (NPI Social and NPI Outdoor), and the proxy for the state of economy (TTMI) are plotted below. NPI: nonpharmaceutical intervention; TTMI: truck toll mileage index.

All domains showed pronounced changes in people’s search behavior in the corresponding period from January 6 to September 1, 2020, although the changes were mostly short-lived (Figures 2 and 3). Many domains showed different onsets during the COVID-19 crisis. Disease- and virus-related domains (ie, *pandemic disease*, *health care institutions*, *infection*, *symptoms*, *testing*, *hand hygiene*, *disinfectant*, *parenting and childcare*, *COVID-19 restrictions,* and *economic impact)* show an early onset, but searches decreased quickly. Public interest in political leaders peaked multiple times, while the domains *vaccination*, *social life*, *homeschooling*, *hobbies and sports*, *psychosocial impact,* and *dispatching* seem to be of higher, long-term interest. Interest in other domains seemed to be related to the onset of the NPIs (eg, the domain *mask* peaked when the obligation to wear masks in shops and public transport was announced). Interest in *government support* (ie, financial support programs provided by the national government) increased in later stages of the lockdown, while *COVID-19 relaxations* and *conspiracy theories* attracted attention primarily in the middle of the lockdown phase. For each domain, noticeable changes in the level (ie, mean for a chosen period of time) indicate significant changes in public interest during the examined lockdown period from March 8 to June 26, 2020, in Germany.

### Time Series Regression Analysis

We conceptualized search interests in all 27 domains as criterion variables in ARIMA regression analyses and entered four predictors—namely, daily new infection cases, both NPI factors (NPI Outdoor and NPI Social), and the TTMI as a proxy for the economic state. All analyses were conducted for Germany as a whole, as low ICCs indexed no meaningful heterogeneity between federal states. Table 3 summarizes the results for the regression part. Overall, all predictors showed a different pattern and, thus, predictive value for different domains. Although new infections seemed to have a delayed effect on public interest (see the “Lead” column), the NPI factors and TTMI had their highest effect directly at lag 0. Almost all models benefitted from the ARIMA approach, as shown in Table 4. Most domains needed differencing to account for nonstationarity. Additionally, to handle the correlated residual structure, almost all domains were estimated with an autoregressive or moving average component.

**Table 3:** ARIMA regression results for the regression part with standardized betas for the intercept and all relevant predictors (infection cases, both NPI factors, and the TTMI), separate for all domains^a^.

Domain	Regression term														
	New infections				NPI Outdoor				NPI Social				TTMI		
	Lead	β_1_	*P* value	Lead		β_2_	*P* value	Lead		β_3_	*P* value	Lead		β_4_	*P* value
Pandemic disease	–12	.15	.03	0		–.22	.21	0		–.26	.09	0		–.02	.27
Health care institutions	–9	.23	.02	0		.25	.07	–13		.60	<.001	–13		–.09	.03
Political leaders	–5	.26	.16	0		.01	.99	–11		.26	.40	–11		–.18	.06
Infection	–7	.09	.24	0		.14	.40	–12		.61	<.001	–12		–.05	.09
Symptoms	–11	.10	.03	0		.03	.82	0		–.47	<.001	0		–.03	.17
Information seeking	–11	.07	.58	0		.38	.24	0		–.96	<.001	0		–.08	.04
Testing	–13	.08	.58	0		–.09	.76	0		–.27	.33	0		–.11	.03
Hand hygiene	–13	.24	.04	0		–.17	.37	0		–.21	.22	0		–.03	.52
Mask	–18	–.03	.86	–21		.77	.21	0		–.04	.87	0		–.06	.03
Disinfectant	–12	.03	.77	0		–.04	.85	0		–.21	.32	0		.01	.90
Convenience goods	–4	.18	.04	0		.29	.01	–9		.42	<.001	–9		–.06	.06
Vaccination	–5	.05	.72	0		–.33	.16	–13		1.18	<.001	–13		–.05	.44
Parenting and childcare	0	–.32	.02	0		–.25	.18	0		–.17	.42	0		.07	.38
COVID-19 restrictions	–6	.07	.27	0		.44	.02	–10		.57	<.001	–10		–.04	.19
COVID-19 relaxations	–1	–.13	.35	–2		.65	<.001	–15		.18	.70	–15		.02	.56
Economic impact	–1	.48	<.001	–10		–.05	.94	–8		.36	.07	–8		–.02	.70
Sexual interest	–12	–.12	.58	0		.24	.47	0		.05	.86	0		.16	.10
Social life	–4	.21	.15	–10		–.49	.18	–3		.57	<.001	–3		–.12	.07
Homeschooling	–18	–.07	.75	0		.49	.27	0		.26	.48	0		–.17	<.001
Business communication	0	.34	<.001	–10		–.09	.88	–4		.34	.06	–4		–.09	.06
Hobbies and sports	0	–.16	.14	–10		–.04	.93	–3		1.07	<.001	–3		–.05	.27
Renovation	–11	–.68	<.001	0		.01	.99	0		.06	.68	0		–.01	.91
Online shopping	–3	.30	<.001	0		.47	<.001	–9		.49	<.001	–9		–.10	.05
Dispatching	0	.09	.53	0		–.03	.89	0		.27	.19	0		–.43	<.001
Psychosocial impact	–3	.26	.09	0		.29	.11	–9		.40	.02	–9		–.05	.48
Conspiracy theories	–21	–.03	.88	–21		.25	.82	–1		.31	.33	–1		–.07	.40
Government support	–7	.11	.59	0		.19	.28	–13		–.41	.27	–13		.23	.01

^a^Lead corresponds to the forward-shifted time series for the corresponding number of days. Zero values indicate the original time series without shift. ARIMA: autoregressive integrated moving average; NPI: nonpharmaceutical intervention; TTMI: truck toll mileage index.

**Table 4:** ARIMA regression results for the ARIMA model part with standardized betas for the autoregressive and moving average components^a^.

Domain	ARIMA (*p,d,q*)			ARIMA term								
				Autoregressive component					Moving average component			
	*p*	*d*	*q*	AR(1)	*P* value	AR(2)	*P* value	MA(1)		*P* value	MA(2)	*P* value
Pandemic disease	1	1	1	–.94	<.001	N/A^b^		.85		<.001	N/A	
Health care institutions	0	1	1	N/A		N/A		–.64		<.001	N/A	
Political leaders	2	0	0	.32	<.001	.21	.04	N/A			N/A	
Infection	0	1	1	N/A		N/A		–.28		.02	N/A	
Symptoms	1	1	3	.61	<.001	N/A		–1.03		<.001	.40	.04
Information seeking	0	1	0	N/A		N/A		N/A			N/A	
Testing	0	1	1	N/A		N/A		–.26		.03	N/A	
Hand hygiene	0	1	1	N/A		N/A		–.57		<.001	N/A	
Mask	1	1	1	–.59	<.001	N/A		.85		<.001	N/A	
Disinfectant	0	1	0	N/A		N/A		N/A			N/A	
Convenience goods	0	1	2	N/A		N/A		–.53		<.001	–.22	.06
Vaccination	0	1	1	N/A		N/A		–.52		<.001	N/A	
Parenting and childcare	0	0	1	N/A		N/A		.40		<.001	N/A	
COVID-19 restrictions	1	0	2	.97	<.001	N/A		–.41		<.001	.30	<.001
COVID-19 relaxations	1	0	0	.87	<.001	N/A		N/A			N/A	
Economic impact	0	1	1	N/A		N/A		–.50		<.001	N/A	
Sexual interest	0	1	1	N/A		N/A		–.70		<.001	N/A	
Social life	0	1	2	N/A		N/A		–.57		<.001	–.34	<.001
Homeschooling	0	1	0	N/A		N/A		N/A			N/A	
Business communication	0	1	1	N/A		N/A		–.48		<.001	N/A	
Hobbies and sports	1	1	1	.28	.03	N/A		–.85		<.001	N/A	
Renovation	1	0	0	.41	<.001	N/A		N/A			N/A	
Online shopping	0	1	1	N/A		N/A		–.68		<.001	N/A	
Dispatching	1	0	0	.56	<.001	N/A		N/A			N/A	
Psychosocial impact	0	1	1	N/A		N/A		–.89		<.001	N/A	
Conspiracy theories	0	1	1	N/A		N/A		–.46		<.001	N/A	

^a^The ARIMA model parameters *p*, *d*, and *q* represent the order of the autoregressive components, the order of differencing of the original time series, and the order of the moving average components, respectively. ARIMA: autoregressive integrated moving average.

^b^N/A: not applicable.

## Discussion

### Principal Results

This study was conducted to test possible effects of the COVID-19 pandemic, in conjunction with lockdown NPIs, daily new infections, and the state of the national economy, on people’s social and psychological interests, going beyond previous research examining only a few domains for a limited timespan [33,35-37]. Specifically, we tested search interests for 27 broad domains capturing social life and psychological functioning. To this end, 249 search terms from the Google Trends database were used as observed indicators. Factor scores obtained in time series factor analyses were used to overcome the challenges of unreliability and specificity of single indicator variables. Search interests in all investigated domains were found to be heavily affected by the COVID-19 pandemic. Confirming previous findings, increases in search interests were mostly short-lived [18,29], indicating possible knowledge translation gaps, which could be overcome by adapting public information strategies [23-25].

While disease- and virus-associated domains (ie, *pandemic disease, health care institutions, infection, symptoms, hand hygiene, disinfectant,* and *COVID-19 restrictions*) showed early increases in public interest, the domains *homeschooling* and *mask* were not triggered by first reports but peaked later, when the lockdown measures had been issued for some time already. Searches for the names of *political leaders* peaked multiple times, whereas the domains *vaccination*, *social life*, *hobbies and sports*, *psychosocial impact,* and *dispatching* revealed more long-term interest. This is in line with previous research, which has shown that people responded to the first reports of infections by seeking out information about COVID-19, as measured by searches for coronavirus, coronavirus symptoms, and hand sanitizer [30]. Contrary, personal health strategies (ie, mask, medication) and community-level policies (ie, school closures, testing) were found to peak at later stages of the pandemic [30]. Additionally, differences between domains and their delayed occurrence have been reported multiple times [19,30,46].

We did not find meaningful differences between German federal states for any of the studied domains. This corresponds with high correlations for the single search term “coronavirus” for multiple countries in previous research [29]. However, other research has shown differences in the onset of COVID-19 search terms in Google Trends, even for subregions within countries [18]. Hence, we assume that the highly comparable search interests observed across German federal states could reflect, in part, that German federal states issued comparable clusters of NPIs simultaneously [5].

Generally, cross-correlations between domains were found to be highest for nonshifted time series, which we tentatively interpret as indicating that the announcement of NPIs immediately influences public interest. This potentially reveals a decisive point worth considering when developing information strategies.

Change point analyses indicate level changes in public interest over the lockdown period for all domains. However, there was no conclusive evidence that change points for domains consistently coincide with the onset of the NPIs. This suggests that changes observed in search interests for domains are not directly related with the onset of NPIs. Nevertheless, certain domains characterized by a high cross-correlation (eg, *pandemic disease* and *health care institutions*) revealed a comparable pattern of change points (see Figure 1 for all cross-correlations and Figures 2 and 3 for the time series with change points). Change points may thus be prone to changes within and between time series but are not sensitive enough to coincide with the onset of new infection cases or the onset of NPIs. They could provide circumstantial evidence of co-occurrence of events, but they lack sensitivity to identify temporarily lagged effects.

To shed light on possibly lagged effects, all domains were subject to time series regression. In line with previous evidence [32,36,37] that search interests for COVID-19–related search tags follow increases in infection rates after 8-14 days, daily new infections were a leading predictor in the current study as well. This confirmed a comparably lagged pattern, especially for the domains *pandemic disease*, *health care institutions*, *symptoms,* and *hand hygiene* within the aforementioned lead (see Table 3). A rise in new infection cases also predicted search interest for *convenience goods*, *economic impact,* and *business communication* but was related to reduced interest in *parenting and childcare* and *renovation*. As the lockdown has been shown to considerably affect social life and people’s routines [25,46,47], we chose the two NPI factors capturing regulations pertaining to outdoor activities and social life as additional predictors. Both factors appeared to be differentially related with domains. The factor capturing regulation of outdoor activities was positively related with search interests for *convenience goods*, *COVID-19 relaxations,* and *online shopping*. The factor capturing regulation of social activities predicted search interests with the same direction for the same domains as the factor capturing regulation of outdoor activities, and additionally *health care institutions*, *infection*, *vaccination*, *social life*, *hobbies and sports*, and *psychosocial impact*. Further, it was negatively related with the domains *symptoms* and *information seeking*. Overall, our results indicate that NPIs targeting the regulation of social life may have more impact on people’s behavior and routines.

The proxy variable for the state of economy (TTMI) revealed relatively consistent negative relations with the investigated search interests of arguably low magnitude. The direction of this effect is plausible, as it indicates increasing interest as the economy declines. However, the small magnitude of these effects suggests that the economic situation had a rather small impact on people’s social and psychological interests in the first lockdown period.

### Limitations

This study used the information available in public databases and Google Trends as a proxy for people’s psychological interests, motives, and concerns. Given that inferences were drawn only from individuals with access to the internet who use the Google search engine, results could be biased to some extent (eg, because older adults or low-income people could be underrepresented in the sample). However, we deem this effect to be rather small, as 95% of households in Germany have access to the internet [60]. Further, there is evidence showing that information drawn from different search engines and social media platforms is highly comparable [36], hence, results can be expected not to be specific for the Google search engine only.

We investigated whether changes in search interests were affected by the onset of the pandemic and the corresponding lockdown in Germany. However, in a globally connected world, news from abroad could have easily affected search interests. In fact, search interest in COVID-19 reached its first peak before infection rates rose in Europe, especially when infection rates rose exponentially in China [32], suggesting a global influence [18]. The fact that regions with the highest numbers of cases were not always the first in terms of spreading information suggests a considerable role of cross-effects [19]. Consequently, regional, national, and even global events can exert an effect on public interest [31].

Some of the used search tags (eg, diarrhea) may not be uniquely related to the COVID-19 outbreak [11]. We addressed this challenge in two ways. First, we investigated relatively long baseline phases to test if interest after the arrival of the pandemic increased meaningfully compared to its baseline. Second, we did not base our analyses on single search tags that could easily be affected by third factors. Rather, we estimated interest in domains based on the aggregated information across several search tags.

Regression analysis revealed the necessity to account for correlated residuals during the linear modelling of Google Trends data. Another alternative for time series data would be the usage of vector autoregressive models (VAR) for prediction. VAR offers the advantage that criterion and predictor variables are interchangeable, allowing the use of all variables without prior assumptions about causality. This seems appropriate as cross-correlation functions in Multimedia Appendix 5 show moderate to high correlations between all studied time series. Even predicting new infection hot spots seems possible by analyzing public interest, given a proper sample size. Although this might be counterintuitive, there is supporting evidence that Google Trends data can be used to find new symptoms caused by diseases [41].

Although we z-standardized all variables prior to regression analysis, some betas exceeded the value of 1 (see Table 2, predictor NPI Social for the domains *vaccination* and *hobbies and sports*), possibly indicating suppression effects. However, post hoc correlation analysis between predictors indicated only moderate positive correlation. Thus, predictors are not highly collinear and betas exceeding 1 may reflect computational imprecision.

In line with our preregistration [49], our study was multivariate and multiple analyses had to be conducted. However, we did not correct statistical significances for multiple testing as we believe that effect sizes of the observed relations are more meaningful and closely in line with previous research for empirically derived domains. Nonetheless, we consider replication in independent samples very valuable to corroborate the obtained results, especially for the rationally selected domains used in the exploratory analyses.

### Conclusions

This study shows that people’s search interests are considerably affected by the pandemic and the issued NPIs. Increases were observed across a broad set of domains, reflecting psychological interests, motives, and concerns. For disease- and virus-associated domains, the increases occurred shortly after the onset of the pandemic, whereas domains capturing personal health strategies or community-level policies peaked later during the lockdown period. *Renovation* was the only domain to show an early decrease in public interest. The different onsets of the studied domains may indicate that some consequences are yet to become apparent, (eg, an increase in mental disorders due to novel life circumstances during the lockdown). Further research could, therefore, tap into multiple, more detailed domains using the demonstrated approach of TSFA while combining multiple keywords to handle undesirable error variance.

Using Google Trends data provides insights into people’s search interests. The high sensitivity backs the interpretation of changes in public interest as a behavioral tendency [18,46]. Analyzing Google Trends data solves the problem of data scarcity, providing inexpensive information regarding possible behavioral changes that would be difficult to attain by means of questionnaire and survey data. This study shows pronounced changes in people’s search interests during crises and lockdown, although prediction of specific developments is moderate at best. Given the relatively broad effects across the diverse domains, manifold effects on psychological interests, motives, and worries are indicated. In turn, these effects can help policy makers to develop a better insight into people’s needs and concerns and to adapt and develop suitable information, prevention, and intervention strategies.

## Appendix

Multimedia Appendix 1 Overview of domains and corresponding search tags. Search tags we could not retrieve via Google Trends are marked with a *. Search tags that were dropped during data reduction and preprocessing are marked with a †.

Multimedia Appendix 2 Map showing the 16 German federal states colored by 7-day incidence of COVID-19 per 100,000 inhabitants for the middle (April 30, 2020) and the end of the lockdown (June 26, 2020) in Germany.

Multimedia Appendix 3 Summary statistics for population number and density, as well as cumulative infection and incidence rates for all federal states and Germany.

Multimedia Appendix 4 Scree plots for time series factor analysis for each domain after deleting items with negative loadings, in line with the preregistered protocol. All scree plots support the predicted underlying one-factor structure, indicating the communality of the respective domain.

Multimedia Appendix 5 Cross-correlation functions for each predictor for leads and lags of up to ±21 days for all domains used in the analysis. Predictive value as a function of lead/lag is given for infection rates, the countermeasure factors capturing regulation of outdoor activities (CM Outdoor) and regulation of social life (CM Social), and the truck toll mileage index as an indicator of the state of economy in Germany.

## Abbreviations

API: advanced programming interface
ARIMA: autoregressive integrated moving average
ICC: intraclass correlation
NPI: nonpharmaceutical intervention
NPI Social: NPI factor regulating social life
NPI Outdoor: NPI factor regulating outdoor activities
RKI: Robert Koch Institute
TSFA: time series factor analysis
TTMI: truck toll mileage index
VAR: vector autoregressive models
WHO: World Health Organization
ZPID: Leibniz Institute for Psychology
