# Supplementary material for: Interests, Motives, and Psychological Burdens in Times of Crisis and Lockdown: Google Trends Analysis to Inform Policy Makers
Source: J Med Internet Res. 2021 Jun 1;23(6):e26385. doi: 10.2196/26385 (PMC8171287; doi:10.2196/26385)
Supplement: Multimedia Appendix 1 [file jmir_v23i6e26385_app1.docx]

**Multimedia Appendix 1**

This is a Multimedia Appendix to a full manuscript published in the J Med Internet Res. For full copyright and citation information see <http://dx.doi.org/10.2196/26385>

Overview of domains and corresponding search tags. Search tags we could not retrieve via Google Trends (GT) are marked with an *. Search tags which were dropped during the data reduction and pre-processing are marked with a †.

| **pandemic disease** | **Health care**  **institutions** | **political leaders** | **infection** | **symptoms** | **information**  **seeking** | **testing** | **hand hygiene** | **mask** | **disinfectant** | **convenience goods** | **vaccination** | **parenting and**  **child care** |
| --- | --- | --- | --- | --- | --- | --- | --- | --- | --- | --- | --- | --- |
| corona  (corona) | WHO  (WHO) | merkel^e^ | infektion  (infection) | symptome corona  (symptoms corona) | nachrichten  (news) | corona test  (corona test) | hände waschen  (wash hands) | masken  (masks) | desinfektionsmittel  (disinfectant) | backhefe  (yeast) | corona impfung  (corona vaccination) | kindergarten  (kindergarten) |
| coronavirus  (coronavirus) | robert koch institut  (Robert Koch Institute) | spahn^f^ | infektionen  (infections) | trockener husten  (dry cough) | pressekonferenz  (press conference) | teststellen*  (testing locations) | händewaschen  (wash hands) | nähanleitung maske*  (mask sewing instructions) | desinfektionsgel  (disinfectant gel) | mehl  (flour) | impfen  (vaccinate) | schule  (school) |
| coronaviren  (corona viruses) | rki^a^  (RKI) | söder^g^ | infektionsrate  (infection rate) | fieber  (fever) | corona liveticker  (corona liveticker) | antikörpertest^†^  (antibody test) | händedesinfektion  (hand disinfection) | fahrrad maske*  (bike mask) | desinfektion  (disinfection) | konserven  (canned food) | impfmittel*  (vaccine) | kinderbetreuung  (childcare) |
| norovirus  (norovirus) | johns hopkins  (Johns Hopkins) | laschet^h^ | neuinfektionen  (new infections;  i.e. incidence) | gliederschmerzen  (limb pain) | corona update  (corona update) | schnelltest  (rapid test) | hände desinfizieren  (disinfect hands) | FFP2  (FFP2) | desinfizieren  (disinfect) | klopapier  (toilet paper) | impfpass^†^  (vaccination certificate) | kindertagespflege  (child day-care) |
| noroviren*  (noroviruses) | wieler^b^ | ramelow^i^ | infektionszahlen  (numbers of infections) | halsschmerzen  (sore throat) | corona aktuell  (corona to date) | testkapazität*  (test capacity) | hände reinigen*  (clean hands) | FFP3  (FFP3) | wie desinfiziere ich*  (how to disinfect) | bananenbrot  (banana bread) | herdenimmunität  (herd immunity) | kindertagesstätte  (day-care centre) |
| covid-19^†^  (COVID-19) | schaade^c^ | dreyer^j^ | reproduktionsfaktor*  (reproduction factor) | atemnot  (respiratory distress) | corona newsticker  (corona news ticker) | test bezahlen*  (paying fees  for test) | handgel  (hand gel) | mund nasen schutz  (mouth and nose protection) | brennspiritus  (denatured alcohol) | backen  (baking) | impfpflicht  (mandatory vaccination) | kinder zuhause^†^  (children at home) |
| sars cov 2  (sars cov 2) | bundeszentrale für gesundheitliche aufklärung  (Federal Centre for Health Education) | kretschmann^k^ | r wert^†^  (r nought) | kurzatmigkeit  (shortness of breath) | coronavirus ticker  (coronavirus ticker) | test hausarzt*  (test family doctor) | händewasch lied*  (hand washing song) | mund nasen bedeckung*  (mouth and nose cover) | sterillium  (sterillium) | hamsterkäufe  (panic buys/ hoarding) | impfzwang^†^  (compulsory vaccination) | regenbogen malen^†^  (drawing rainbow) |
| sars 2003  (sars 2003) | bzga^d^  (BZgA) |  | aerosole^†^  (aerosols) | durchfall  (diarrhoea) | corona ticker  (corona ticker) | abstrich  (smear) | händewasch song*  (hand washing song) | mundschutz  (mouth/ face mask) |  | vorrat  (stock) | zwangsimpung*  (compulsory vaccination) | malen nach zahlen^†^  (colour by numbers) |
| covid sars*  (covid sars) |  |  | ansteckung  (infection) | geruchssinn  (sense of smell) | corona zahlen  (corona numbers) | antikörper  (antibody) | handschuhe  (gloves) | maske oder schutzschild  (mask or protective shield) |  | prepper  (prepper) |  |  |
| wuhan  (wuhan) |  |  | heinsberg studie  (COVID-19 Case-Cluster-Study in Germany) | geschmackssinn  (sense of taste) |  |  |  | stoffmasken  (cloth mask) |  |  |  |  |
| wuhan virus^†^  (wuhan virus) |  |  | corona studie  (corona study) | wie lange dauert corona  (how long lasts; i.e. duration of corona) |  |  |  | gesichtsmasken  (face masks) |  |  |  |  |
| schweres akutes atemwegssyndrom*  (severe acute respiratory syndrome) |  |  | risikopatienten  (high-risk patients) | wann zum arzt corona*  (when to see a doctor corona) |  |  |  | einwegmasken  (single-use mask) |  |  |  |  |
|  |  |  | risikogruppe  (risk group) |  |  |  |  | maske und brille*  (mask and glasses) |  |  |  |  |
|  |  |  | virologe  (virologist) |  |  |  |  | maske und heuschnupfen  (mask and hay fever) |  |  |  |  |
|  |  |  | drosten^l^ |  |  |  |  | maskenpflicht  (obligation to wear a face mask) |  |  |  |  |
|  |  |  | streeck^m^ |  |  |  |  |  |  |  |  |  |
|  |  |  | ischgl^n^ |  |  |  |  |  |  |  |  |  |
|  |  |  | kontaktperson  (contact person) |  |  |  |  |  |  |  |  |  |
|  |  |  | intensivbetten  (intensive care beds) |  |  |  |  |  |  |  |  |  |
|  |  |  | ärtztlicher bereitschaftsdienst  (medical on-call service) |  |  |  |  |  |  |  |  |  |

^a^Abbreviation of Robert Koch Institute; German federal government agency and research institute responsible for disease control and prevention

^b^President of the Robert Koch Institute

^c^Vice-President of the Robert Koch Institute

^d^Abbreviation of the Federal Centre for Health Education

^e^Chancellor of Germany

^f^German Federal Minister of Health

^g^Minister-President of Bavaria

^h^Minister-President of North Rhine-Westphalia

^i^Minister-President of Thuringia

^j^Minister-President of Rhineland-Palatinate

^k^Minister-President of Baden-Württemberg

^l^German virologist

^m^German virologist

^n^Town in Austria which was a major COVID-19 hotspot

| **COVID-10**  **restrictions** | **COVID-19**  **relaxations** | **economic impact** | **sexual interest** | **social life** | **homeschooling** | **Business**  **communication** | **hobbies and sports** | **renovation** | **Online shopping** | **dispatching** | **psychosocial**  **impact** | **conspiracy**  **theories** | **government**  **support** |
| --- | --- | --- | --- | --- | --- | --- | --- | --- | --- | --- | --- | --- | --- |
| lockdown  (lockdown) | lockerungen  (relaxations) | arbeitslos  (unemployed) | dating  (dating) | video app  (video app) | homeschooling  (homeschooling) | videokonferenz software*  (video conferencing software) | online fitness  (online fitness) | parkett  (hardwood floor) | ebay^†^  (ebay) | deutsche post^r^  (German post) | häusliche gewalt  (domestic violence) | china labor*  (china laboratory) | kinderbonus  (child bonus) |
| infektions-schutzgesetz  (infection protection act) | wann öffnen  (when open) | kurzarbeit  (short-time work) | seitensprung  (affair) | facebook  (facebook) | schuljahr  (school year) | MS Teams  (MS Teams) | online yoga  (online yoga) | laminat  (laminate) | ebay Kleinanzeigen^†^  (ebay classifieds) | rücksendung  (return shipment) | hilfetelefon*  (support hotline) | Bill Gates | kindergeld  (child benefit) |
| hygiene-vorschriften  (hygiene regulations) | wie viele personen  (how many people) | lohnausfall*  (loss of wages) | porno  (porn) | instagram  (instagram) | padlet  grundschule^p^*  (padlet elementary school) | skype  (skype) | online casino  (online casino) | tapete  (wallpaper) | amazon  (amazon) | paketverfolgung  (parcel tracking) | jugendamt corona*  (youth welfare office corona) | Bilderberger | konjunktur-  paket  (stimulus package) |
| abstandsregeln^†^  (distance rules) | wie lange  (how long) | darlehen  (loan) | sexstellungen  (sex positions) | tinder^†^  (tinder) | big blue button  (BigBlueButton) | zoom  (zoom) | workout  (workout) | wandfarbe^†^  (wall paint) | lieferservice  (delivery service) | paket  (package) | telefonseelsorge  (crisis helpline) | Freimaurer  (Freemasons) | corona app  (corona app) |
| mindestabstand  (minimum distance) | urlaub 2020  (vacation 2020) | kredit  (loan) | sexspielzeug  (sex toys) | badoo^†^  (badoo) | moodle  (moodle) | krankschreibung telefonisch*  (sick-leave note by phone) | pc spiele  (pc games) | küche  (kitchen) | lieferando  (Just Eat Takeaway) | retoure  (return) | depressionen  (depressions) | New World Order*  (New World Order) |  |
| corona bußgeldkatalog  (corona schedule of penalties) | reisen  (travelling) | miete  (rent) | sex  (sex) | corona trink challenge*  (corona drinking challenge) | abitur^q^ | online meeting  (online meeting) | autokino  (drive-in cinema) | wohnung  (flat) | picnic  (picnic) |  | depression  (depression) | Illuminati |  |
| corona regeln^†^  (corona rules) | gastronomie  (gastronomy) | soforthilfe  (emergency relief) | kondome  (condoms) | gottesdienst online  (church service online) | schulöffnung  (school opening) |  | fernseher  (television) |  | online supermarkt  (online supermarket) |  | isolation  (isolation) | menschenrechte  (human rights) |  |
| besuchen  (visit) | gesichts-behandlung*  (facial) | deutsche bahn corona^o^  (German railway  corona) | one night stand*  (one night stand) |  |  |  | spotify  (spotify) |  | online apotheke  (online pharmacy) |  | einsamkeit  (loneliness) |  |  |
| reisewarnung  (travel warning) | nagelstudio  (nail salon) | fluggesellschaften corona*  (airlines corona) |  |  |  |  | modelleisenbahn^†^  (model railway) |  |  |  | soziale distanz*  (social distancing) |  |  |
| ausgangssperre  (curfew) | kosmetikstudio  (beauty salon) |  |  |  |  |  | stricken  (knitting) |  |  |  | langeweile  (boredom) |  |  |
| nachbarschafts-hilfe  (neighbourly help) | friseur  (hairdresser) |  |  |  |  |  | brettspiele  (board games) |  |  |  |  |  |  |
| quarantäne  (quarantine) | frisör  (hairdresser) |  |  |  |  |  |  |  |  |  |  |  |  |
| Rückholaktion  (return campaign) | spielplatz  (playground) |  |  |  |  |  |  |  |  |  |  |  |  |
| demonstration corona^†^  (protest demonstration corona) |  |  |  |  |  |  |  |  |  |  |  |  |  |

^o^German railway company

^p^Virtual bulletin board

^q^German secondary school certificate

^r^German postal service
