# Supplementary material for: Interests, Motives, and Psychological Burdens in Times of Crisis and Lockdown: Google Trends Analysis to Inform Policy Makers
Source: J Med Internet Res. 2021 Jun 1;23(6):e26385. doi: 10.2196/26385 (PMC8171287; doi:10.2196/26385)
Supplement: Multimedia Appendix 2 [file jmir_v23i6e26385_app2.docx]

**Multimedia Appendix 2**

This is a Multimedia Appendix to a full manuscript published in the J Med Internet Res. For full copyright and citation information see <http://dx.doi.org/10.2196/26385>

Map showing the 16 German federal states coloured by the 7-day incidences per 100 000 inhabitants for the middle (30 April) and the end of the lockdown (26 June) in Germany.


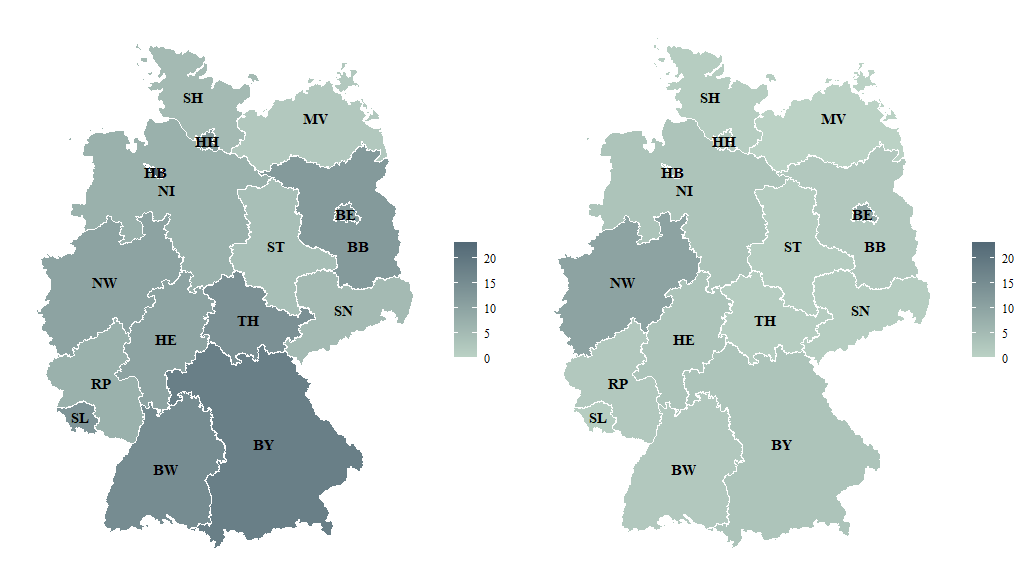


**30 April**

**26 June**
