# Supplementary material for: Interests, Motives, and Psychological Burdens in Times of Crisis and Lockdown: Google Trends Analysis to Inform Policy Makers
Source: J Med Internet Res. 2021 Jun 1;23(6):e26385. doi: 10.2196/26385 (PMC8171287; doi:10.2196/26385)
Supplement: Multimedia Appendix 3 [file jmir_v23i6e26385_app3.docx]

| **Code** | **Federal State** | **Territory**  in km^2^ | **Population**  in Million | **Population density**  per m^2^ | **Cumulative infections** | | | **Incidence** per 100k | | | **7-day Incidence** per 100k | |
| --- | --- | --- | --- | --- | --- | --- | --- | --- | --- | --- | --- | --- |
|  |  |  |  |  | 08 March | 30 April | 26 June | 08 March | 30 April | 26 June | 30 April | 26 June |
|  |  |  |  |  |  |  |  |  |  |  |  |  |
| BW | Baden-Württemberg | 35748 | 11.10 | 311 | 176 | 32290 | 35554 | 1.6 | 291 | 320 | 5 | 1 |
| BY | Bavaria | 70542 | 13.12 | 186 | 120 | 42907 | 48262 | 0.9 | 327 | 368 | 12 | 2 |
| BE | Berlin | 891 | 3.67 | 4090 | 30 | 5941 | 8182 | 0.8 | 162 | 223 | 7 | 3 |
| BB | Brandenburg | 29654 | 2.52 | 85 | 6 | 2923 | 3393 | 0.2 | 116 | 135 | 23 | 3 |
| HB | Bremen | 419 | 0.68 | 1624 | 0 | 900 | 1655 | 0 | 132 | 243 | 10 | 10 |
| HH | Hamburg | 755 | 1.85 | 2446 | 18 | 4852 | 5176 | 1 | 263 | 280 | 10 | 3 |
| HE | Hesse | 21116 | 6.29 | 298 | 13 | 8377 | 10738 | 0.2 | 133 | 171 | 7 | 2 |
| NI | Lower Saxony | 47710 | 7.99 | 168 | 29 | 10338 | 13478 | 0.4 | 129 | 169 | 15 | 2 |
| MV | Mecklenburg-West Pomerania | 23295 | 1.61 | 69 | 5 | 693 | 797 | 0.3 | 43 | 50 | 18 | 3 |
| NW | North Rhine-Westphalia | 34112 | 17.95 | 526 | 342 | 32930 | 42562 | 1.9 | 183 | 237 | 13 | 1 |
| RP | Rheinland-Palatinate | 19858 | 4.09 | 206 | 12 | 6077 | 6974 | 0.3 | 148 | 170 | 11 | 9 |
| SL | Saarland | 2571 | 0.99 | 384 | 1 | 2604 | 2801 | 0.1 | 264 | 284 | 12 | 2 |
| SN | Saxony | 18450 | 4.07 | 221 | 9 | 4696 | 5439 | 0.2 | 115 | 134 | 2 | 0 |
| ST | Saxony-Anhalt | 20457 | 2.19 | 107 | 0 | 1565 | 1868 | 0 | 71 | 85 | 5 | 1 |
| SH | Schleswig-Holstein | 15801 | 2.9 | 184 | 4 | 2702 | 3144 | 0.1 | 93 | 108 | 4 | 1 |
| TH | Thuringia | 16202 | 2.13 | 132 | 1 | 2331 | 3249 | 0 | 109 | 152 | 14 | 1 |
| DE | Germany (Total) | 357581 | 83.17 | 233 | 766 | 162126 | 193272 | 0.9 | 195 | 232 | 11 | 4 |

**Multimedia Appendix 3**

This is a Multimedia Appendix to a full manuscript published in the J Med Internet Res. For full copyright and citation information see <http://dx.doi.org/10.2196/26385>

Summary statistics for population number and density, and cumulative infection and incidence rates for all federal states and Germany
