# Supplementary material for: Interests, Motives, and Psychological Burdens in Times of Crisis and Lockdown: Google Trends Analysis to Inform Policy Makers
Source: J Med Internet Res. 2021 Jun 1;23(6):e26385. doi: 10.2196/26385 (PMC8171287; doi:10.2196/26385)

**Multimedia Appendix 4**

This is a Multimedia Appendix to a full manuscript published in the J Med Internet Res. For full copyright and citation information see <http://dx.doi.org/10.2196/26385>

Scree plots for time series factor analysis (TSFA) for each domain after deleting items with negative loadings, in line with the preregistered protocol. All scree plots support the predicted underlying one-factor structure, indicating the communality of the respective domain.


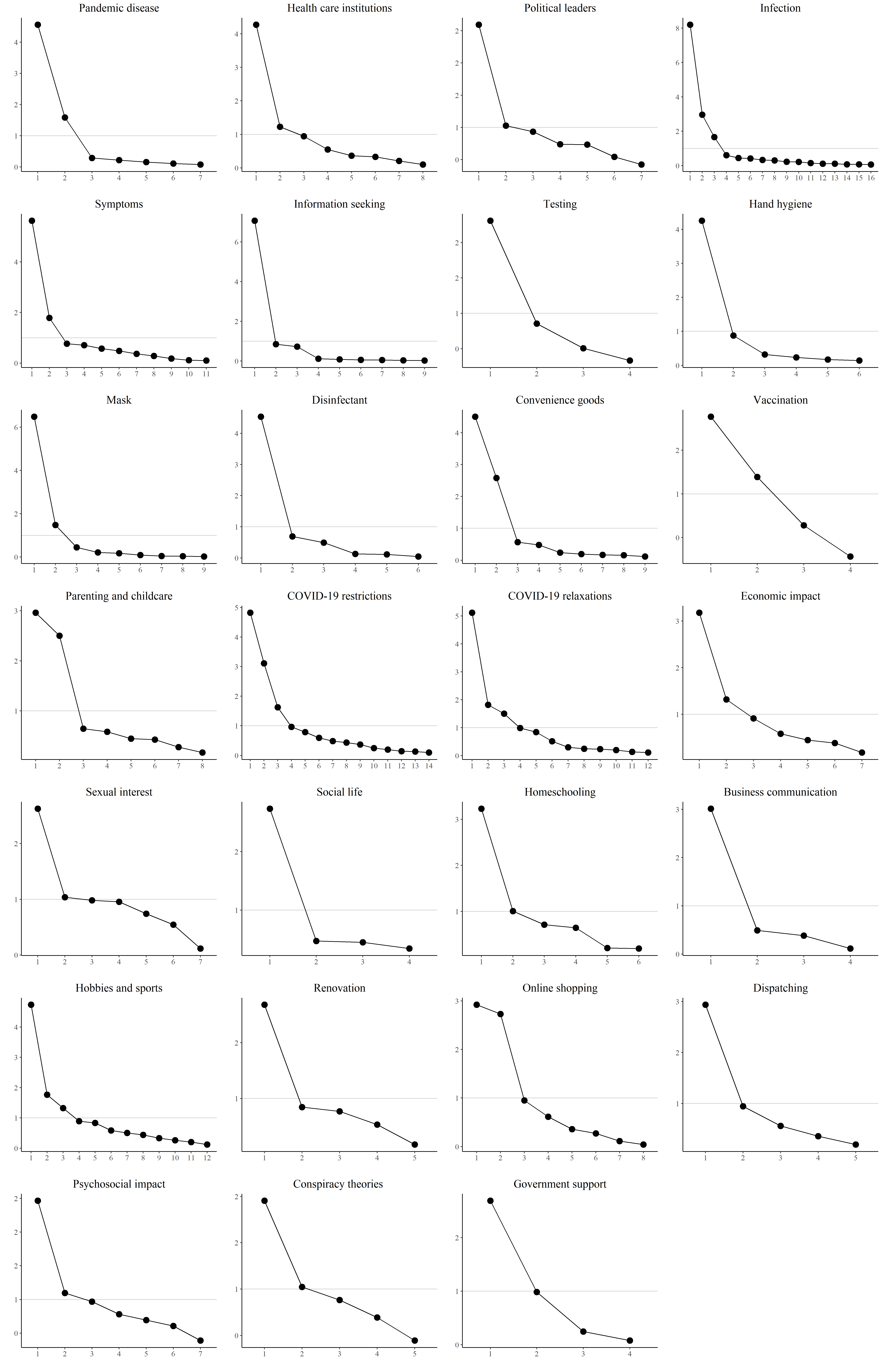

Supplement: Multimedia Appendix 4 [file jmir_v23i6e26385_app4.docx]
