# Supplementary material for: Interests, Motives, and Psychological Burdens in Times of Crisis and Lockdown: Google Trends Analysis to Inform Policy Makers
Source: J Med Internet Res. 2021 Jun 1;23(6):e26385. doi: 10.2196/26385 (PMC8171287; doi:10.2196/26385)

**Multimedia Appendix 5**

This is a Multimedia Appendix to a full manuscript published in the J Med Internet Res. For full copyright and citation information see <http://dx.doi.org/10.2196/26385>

Cross-correlation functions for each predictor for leads and lags of up to ±21 days for all domains used in the analysis. Predictive value as a function of lead/lag is given for infection rates, the NPI factors capturing regulation of outdoor activities (NPI Outdoor) and regulation of social life (NPI Social), and the truck toll mileage index (TTMI) as indicator of the state of the economy in Germany.


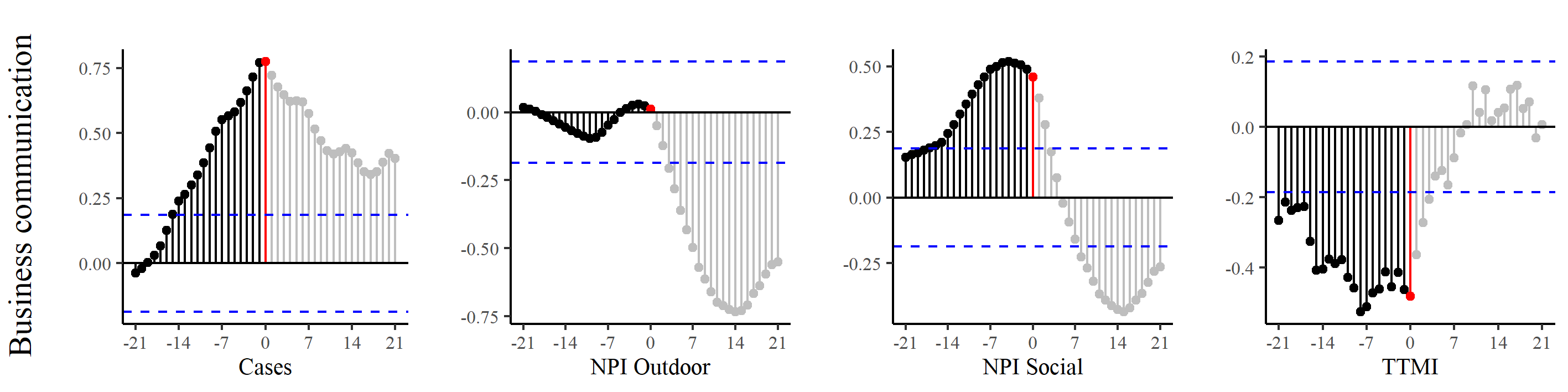

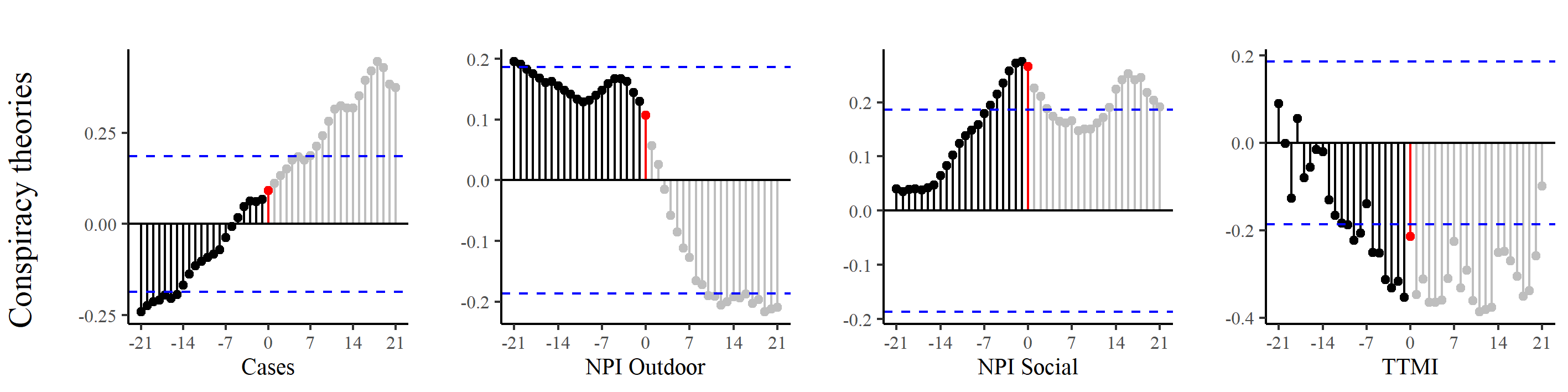

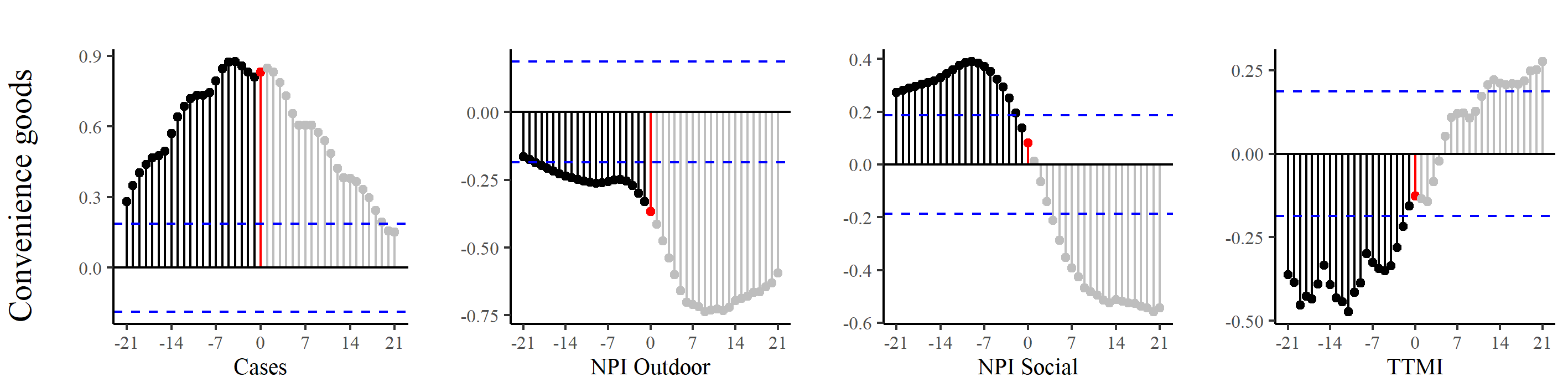

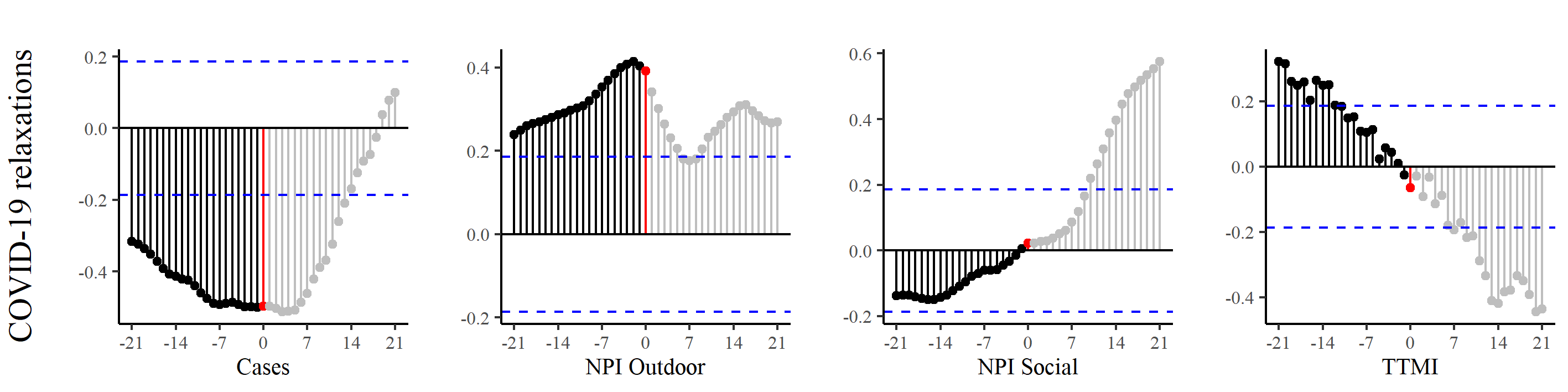

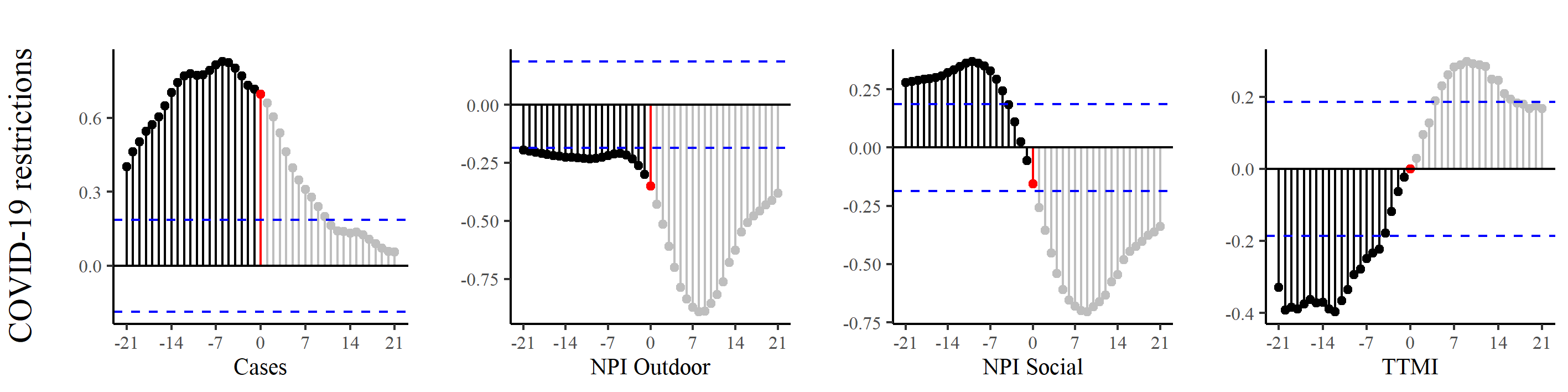

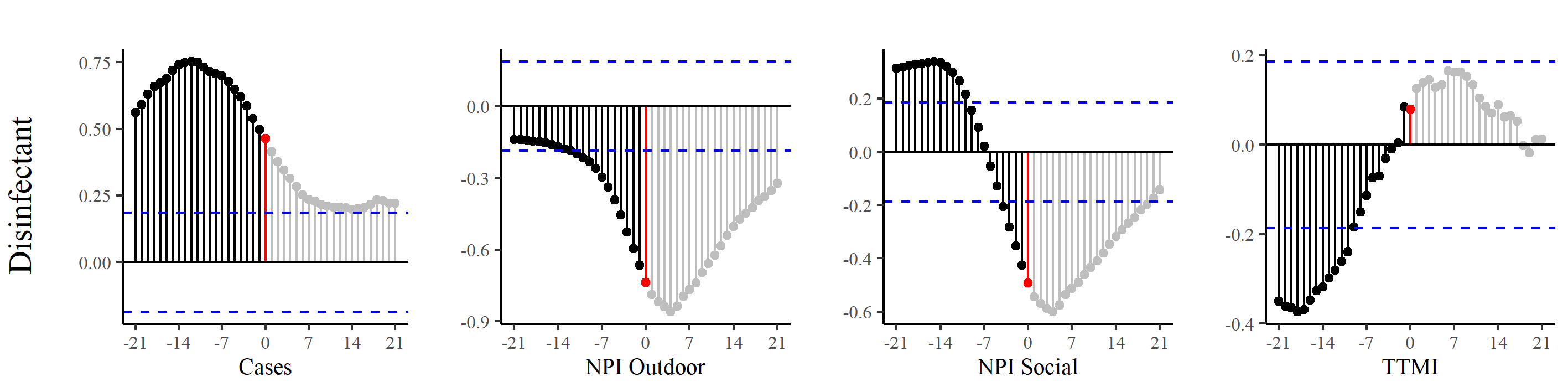

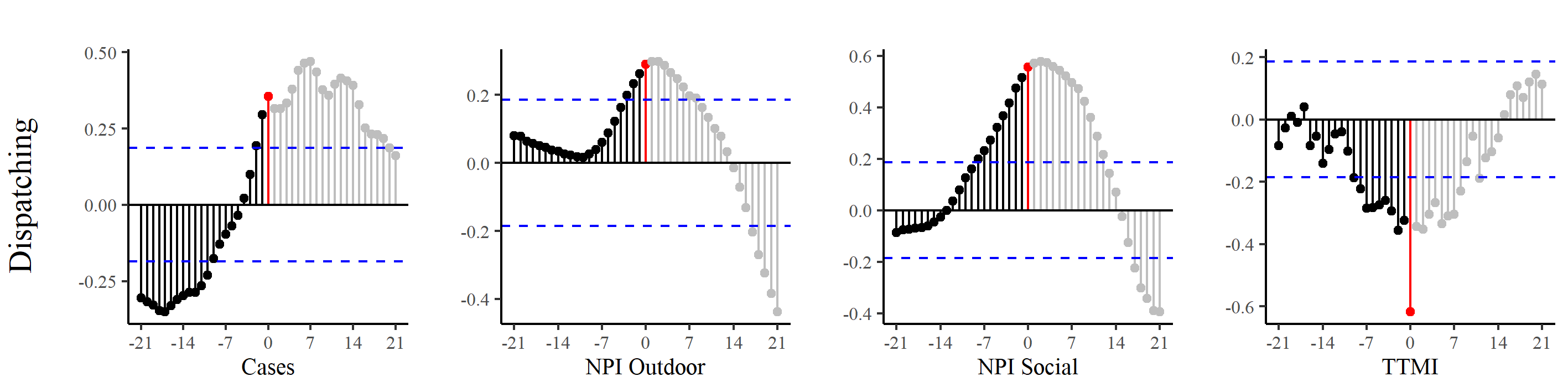

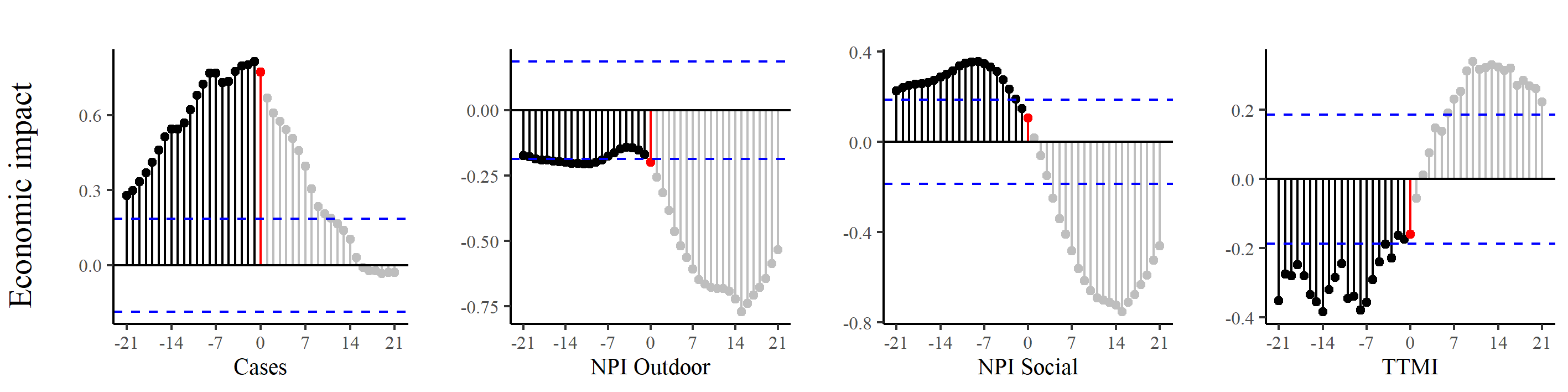

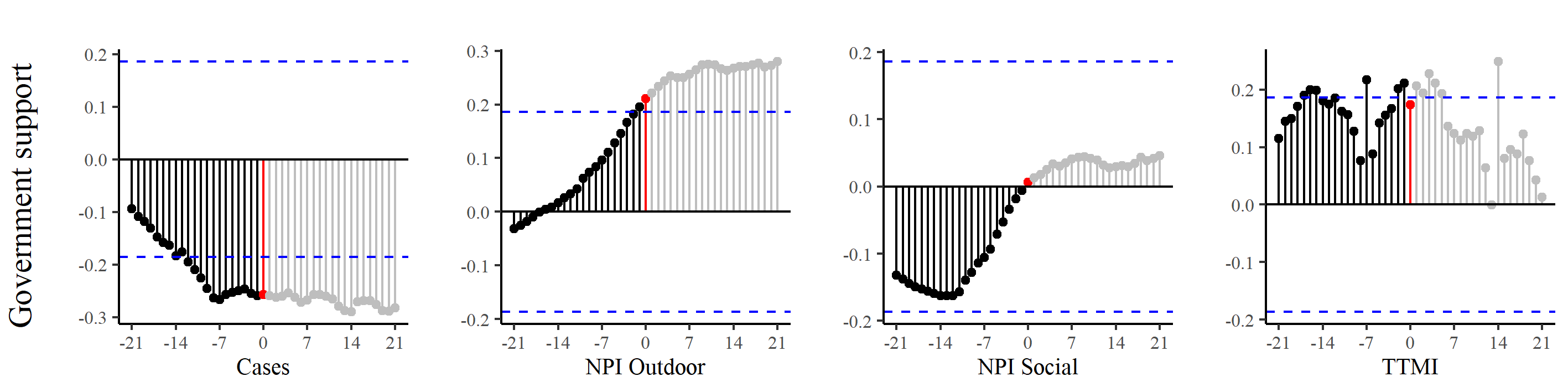

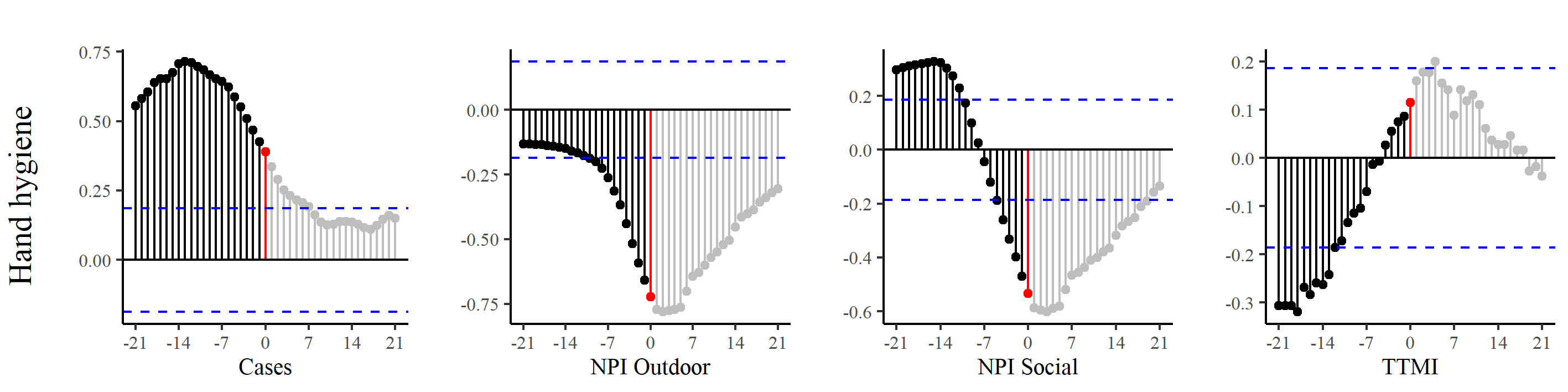

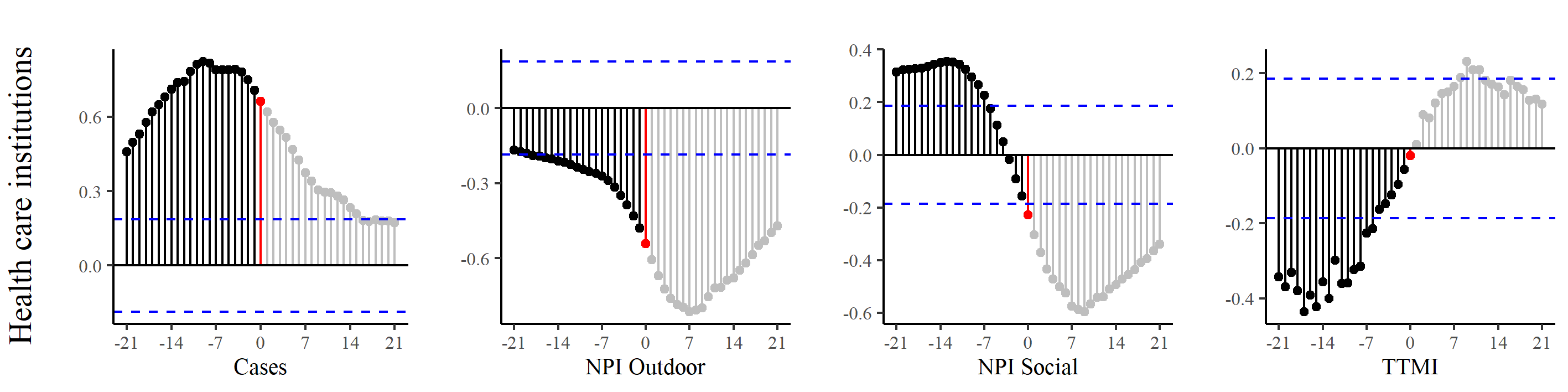

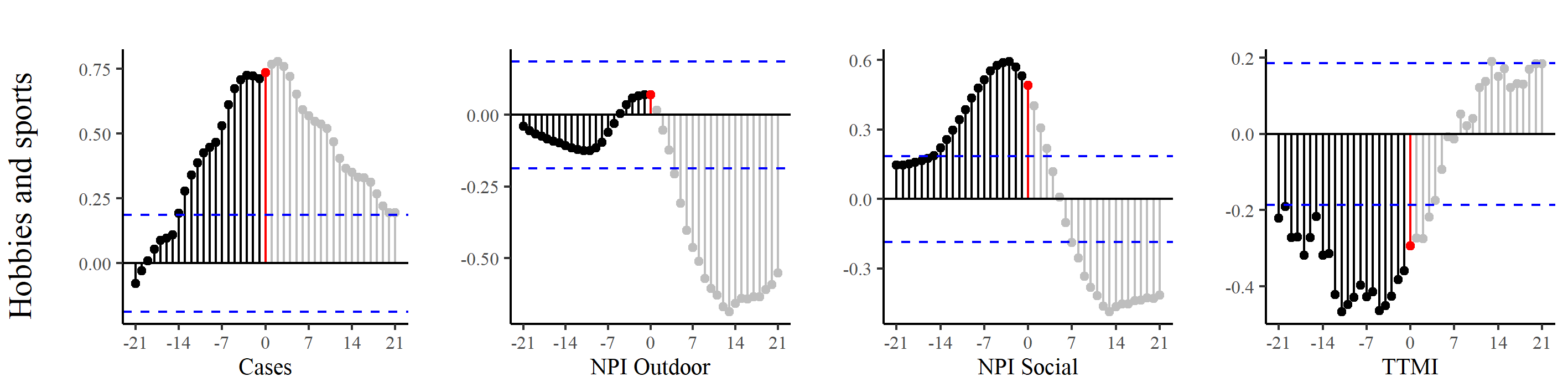

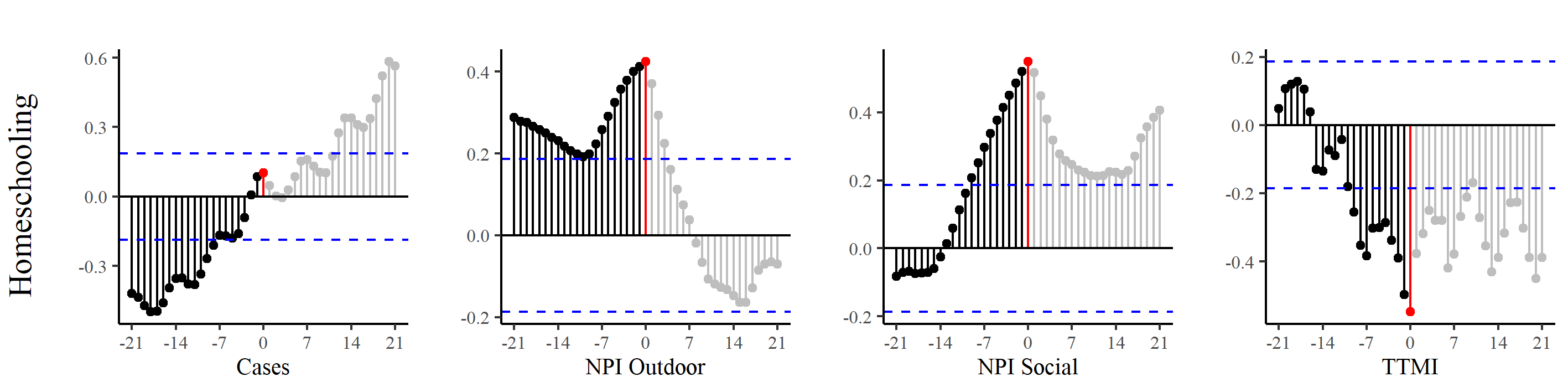

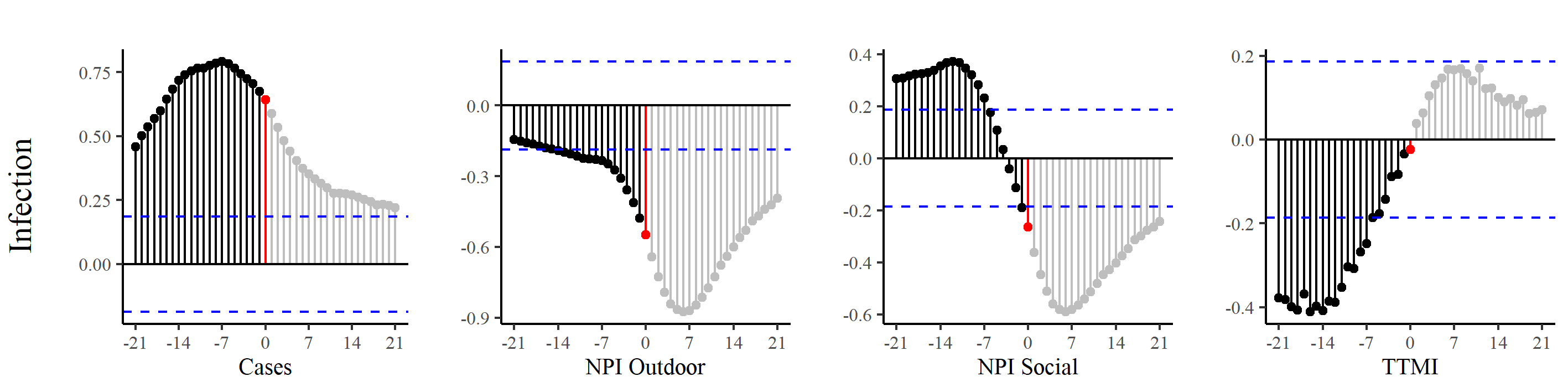

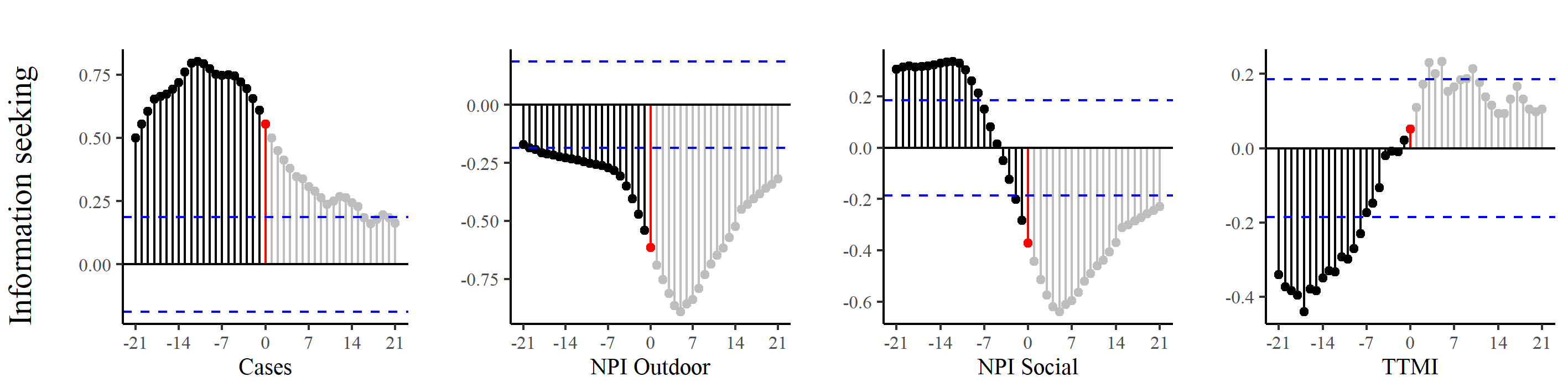

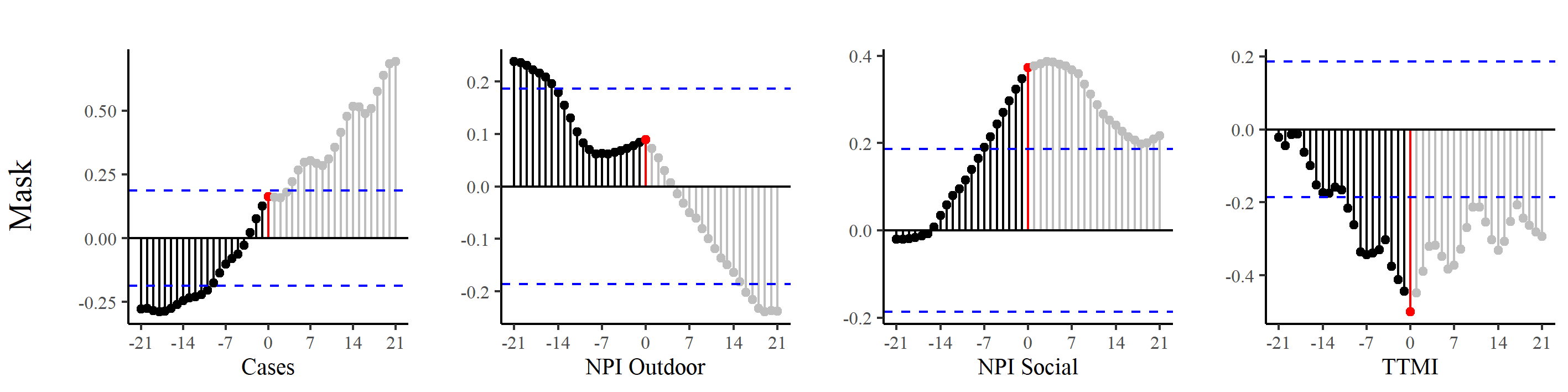

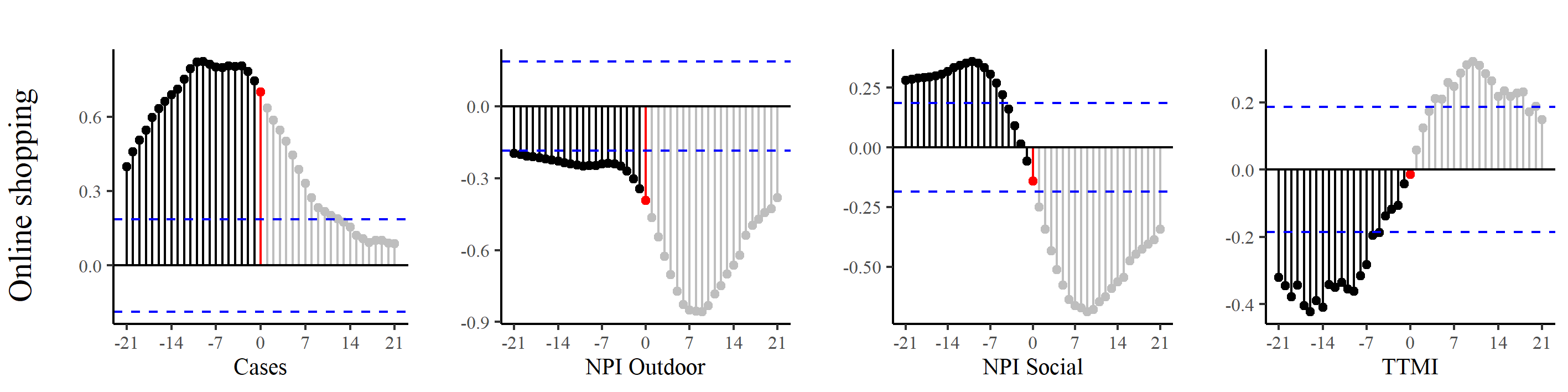

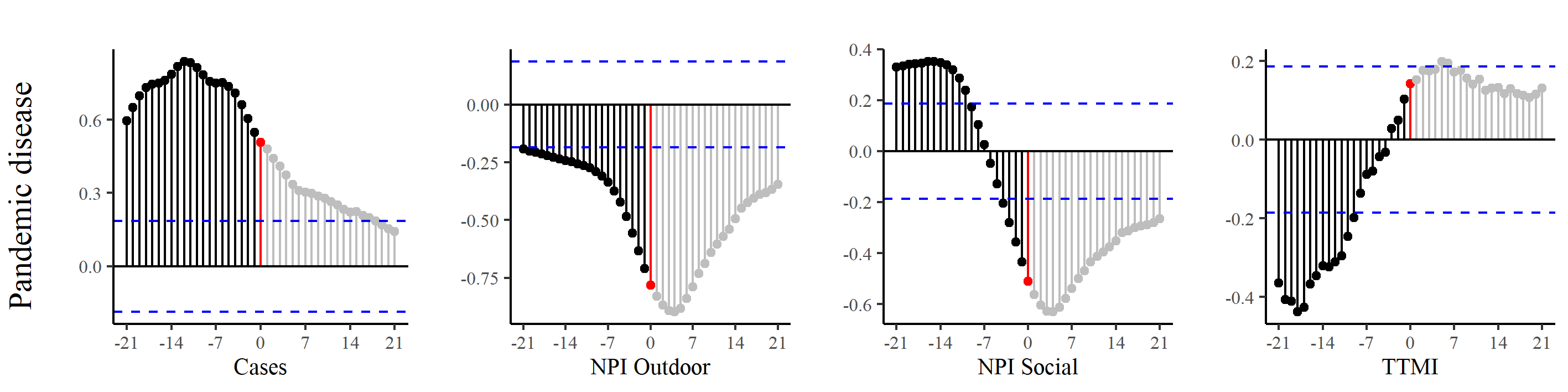

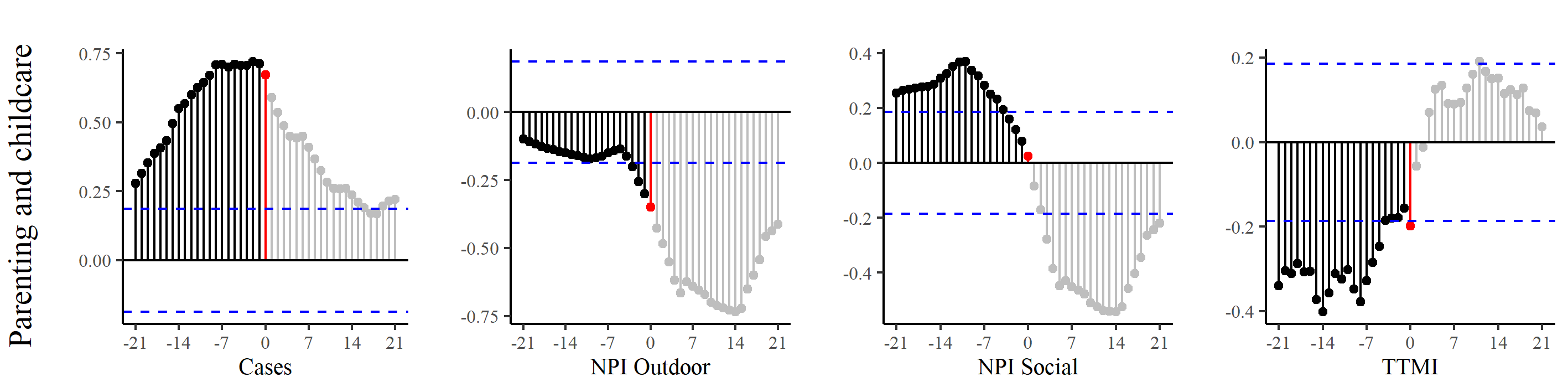

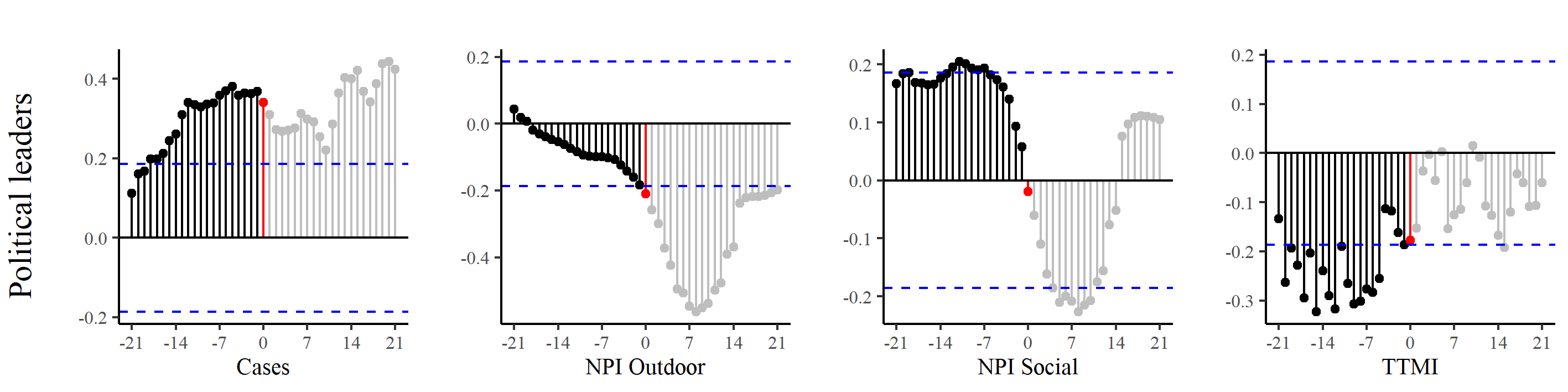

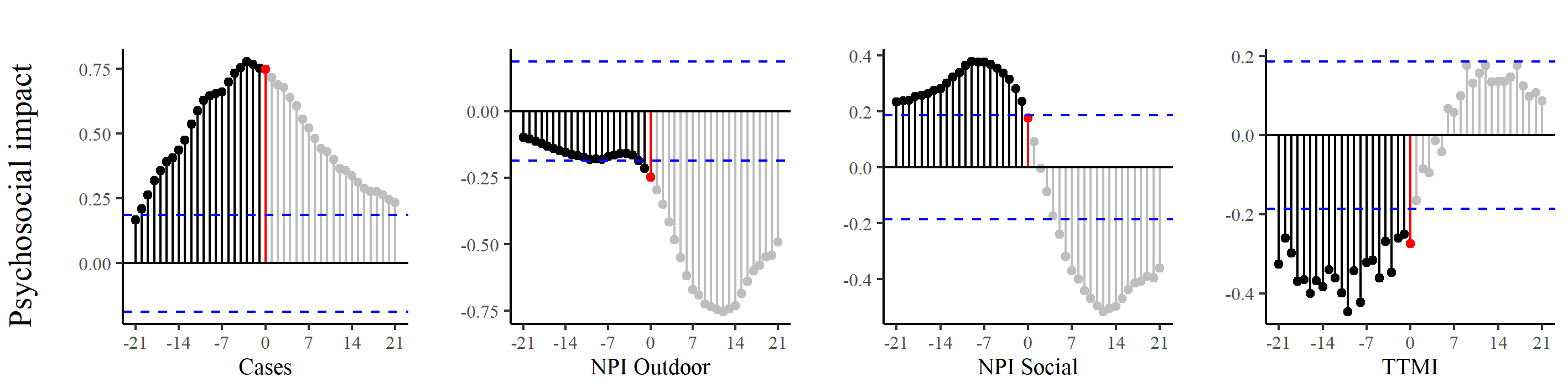

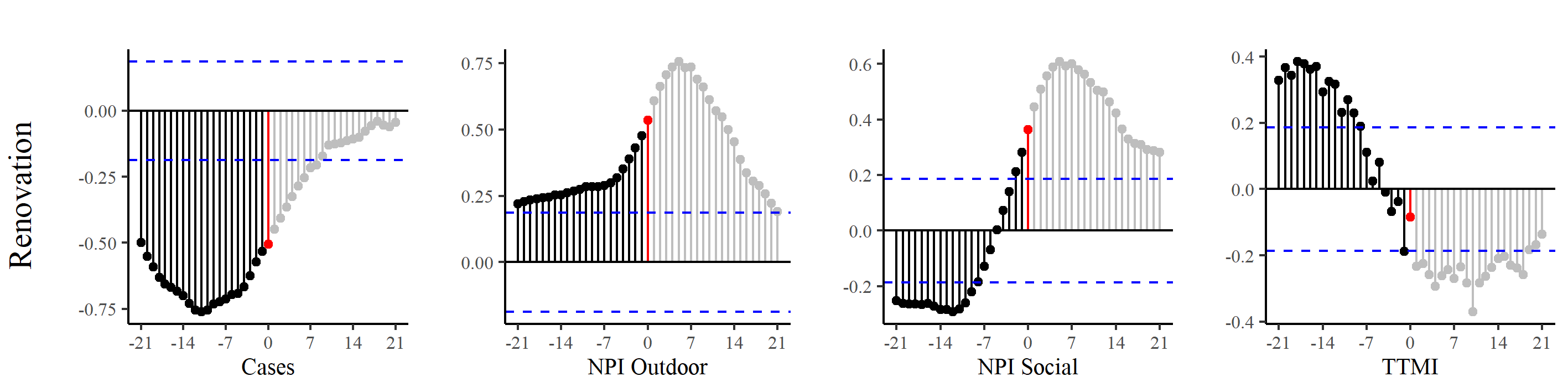

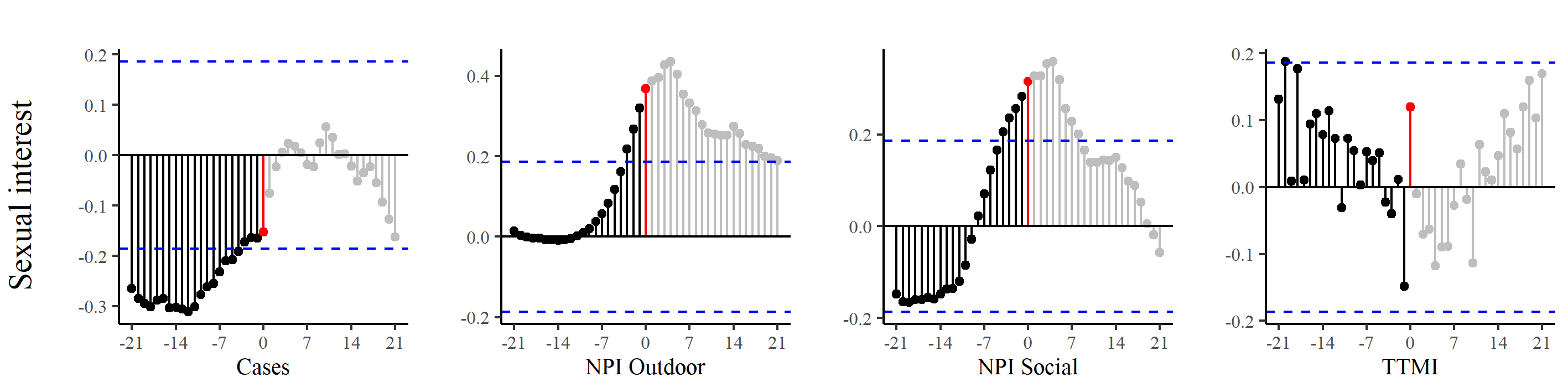

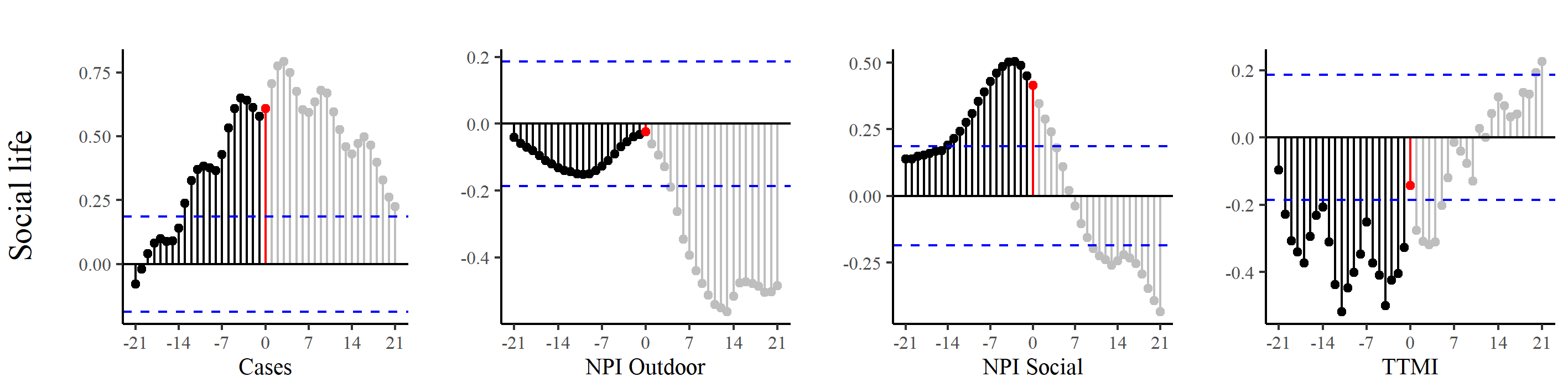

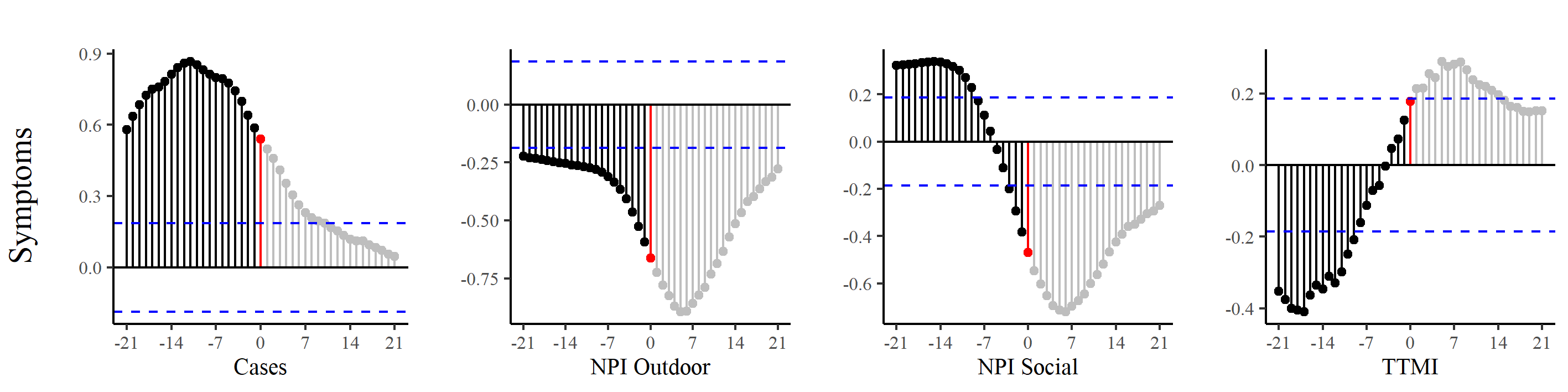

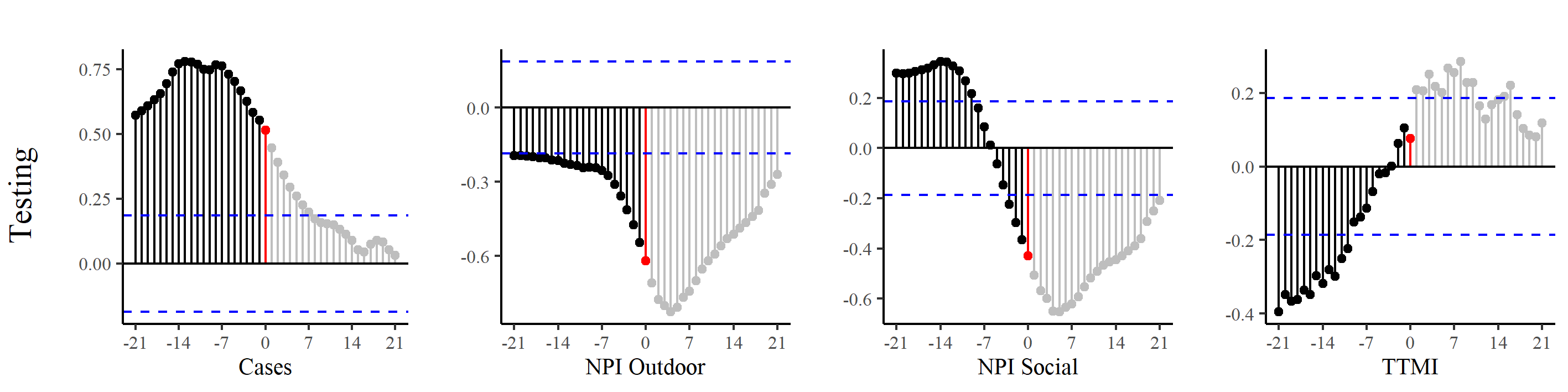

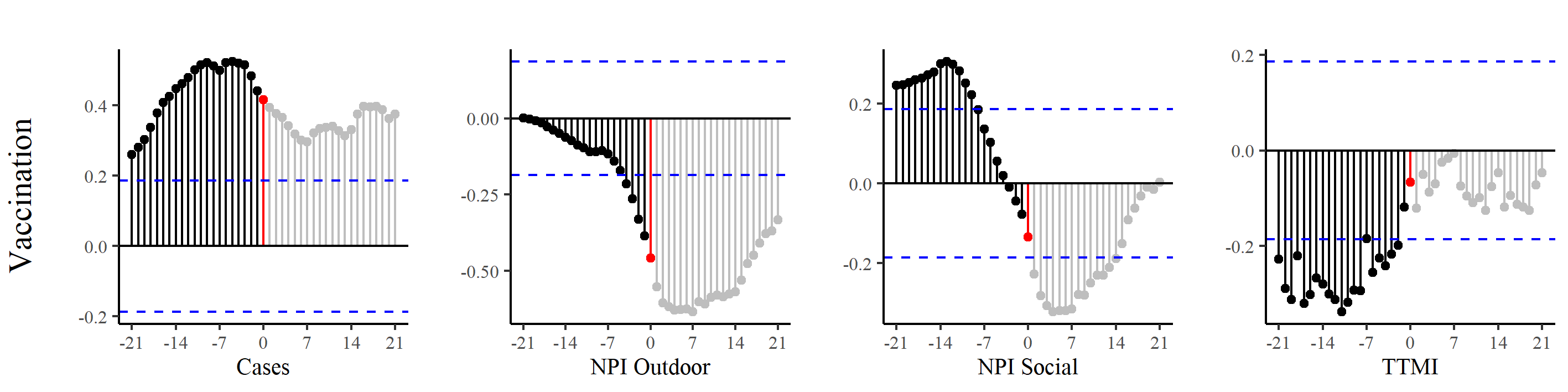

Supplement: Multimedia Appendix 5 [file jmir_v23i6e26385_app5.docx]
